# Identification of Early Warning Signals at the Critical Transition Point of Colorectal Cancer Based on Dynamic Network Analysis

**DOI:** 10.3389/fbioe.2020.00530

**Published:** 2020-05-29

**Authors:** Lei Liu, Zhuo Shao, Jiaxuan Lv, Fei Xu, Sibo Ren, Qing Jin, Jingbo Yang, Weifang Ma, Hongbo Xie, Denan Zhang, Xiujie Chen

**Affiliations:** ^1^College of Bioinformatics Science and Technology, Harbin Medical University, Harbin, China; ^2^School of Stomatology, Harbin Medical University, Harbin, China

**Keywords:** early warning signal, personalized treatment, dynamic network biomarker, colorectal cancer, critical transition point

## Abstract

Colorectal cancer (CRC) is one of the leading causes of cancer-related death worldwide. Due to the lack of early diagnosis methods and warning signals of CRC and its strong heterogeneity, the determination of accurate treatments for CRC and the identification of specific early warning signals are still urgent problems for researchers. In this study, the expression profiles of cancer tissues and the expression profiles of tumor-adjacent tissues in 28 CRC patients were combined into a human protein–protein interaction (PPI) network to construct a specific network for each patient. A network propagation method was used to obtain a mutant giant cluster (GC) containing more than 90% of the mutation information of one patient. Next, mutation selection rules were applied to the GC to mine the mutation sequence of driver genes in each CRC patient. The mutation sequences from patients with the same type CRC were integrated to obtain the mutation sequences of driver genes of different types of CRC, which provide a reference for the diagnosis of clinical CRC disease progression. Finally, dynamic network analysis was used to mine dynamic network biomarkers (DNBs) in CRC patients. These DNBs were verified by clinical staging data to identify the critical transition point between the pre-disease state and the disease state in tumor progression. Twelve known drug targets were found in the DNBs, and 6 of them have been used as targets for anticancer drugs for clinical treatment. This study provides important information for the prognosis, diagnosis and treatment of CRC, especially for pre-emptive treatments. It is of great significance for reducing the incidence and mortality of CRC.

## Introduction

Colorectal cancer (CRC) is one of the leading causes of cancer-related death worldwide (Dienstmann et al., 2017). CRC is mostly asymptomatic until its advanced stages, which contributes to the difficulties of treatment (Aghagolzadeh and Radpour, 2016). Doctors cannot perform routine and effective treatment precisely on patients, such as surgery, radiotherapy and chemotherapy, thus affecting patients’ survival time and quality of life. Therefore, using effective screening methods for cancer is becoming increasingly important for the prevention and inhibition of CRC.

The occurrence and development of cancer are accompanied by the gradual accumulation of somatic mutations. The accumulation of mutations in some critical genes that affect cell proliferation, differentiation and death will eventually lead to cancer. Therefore, finding the mutation order of critical genes in CRC patients and blocking the process in time can effectively prevent the development of cancer and even achieve the goal of pre-emptive treatment. On the other hand, tumor-adjacent tissues are most susceptible to transforming into cancer tissue and will eventually develop into cancer tissue, as the transcriptomes of tumor-adjacent tissue samples often approximate a gene expression signature of invasive cancer, which can be predictive of disease progression in early premalignant lesions (Finak et al., 2008; Graham et al., 2011; Chatterjee et al., 2018). Therefore, this study intends to analyze the process from the level of tumor-adjacent tissues and cancer tissues to find the critical point at which tumor-adjacent tissue transitions to cancer in the process of CRC development, which can serve as an early warning of cancer.

Disease biomarkers are used to diagnose various phases of disease and to monitor severity of disease and response to therapies and can be used to predict prognosis and which patients are likely to respond to therapy (Wang and Ward, 2012). There are already some molecular tumor biomarkers for clinical research, but these biomarkers have limitations in sensitivity and specificity (Kim et al., 2010). CRC patients often show different therapeutic effects and prognoses (Shin et al., 2017). The same biomarker is not effective for all patients with CRC and cancer type and individual differences need to be considered. Therefore, it is necessary to develop CRC-specific, personalized biomarkers for different molecular types and tumor stages, taking the heterogeneity of CRC into account. In this way, the biomarkers being untargetable or producing a poor or no effect due to low sensitivity and specificity will be resolved.

Recently, many studies have found that a sudden change in the state of a system exists in clinical medicine. Such a change often occurs at a critical threshold, or the so-called “tipping point,” at which the system shifts abruptly from one state to another (Chen et al., 2012). During the progression of many complex diseases, the deterioration is not necessarily smooth but abrupt (Chen et al., 2012; Li et al., 2014, 2017; Liu et al., 2014; Mojtahedi et al., 2016; Richard et al., 2016; Lesterhuis et al., 2017). This transformation qualitatively changes the state of the biological system and therefore plays a key role in biological processes. It usually occurs in pre-disease states (or critical states) in the development of complex diseases. The pre-disease state (Achiron et al., 2010) is the limit the normal state can reach before the critical point. At this stage, if properly treated, the disease can be reversed back to normal. Therefore, it is important to determine the critical point before the transition and detect the pre-disease state to prevent the disease with appropriate interventions. The new concept of dynamic network biomarkers (DNBs) is applicable in this type of scenario. It is different from the traditional static method, which was developed on the basis of non-linear dynamics and complex network theory (Chen et al., 2012; Liu et al., 2012). The concept of DNBs fundamentally distinguishes not only normal samples from disease samples but also pre-disease samples from disease samples and thus has great potential to achieve a true warning of cancer. Researchers have applied the DNB method in lung, kidney and thyroid cancers (Liu et al., 2017, 2019) to identify preventative and prognostic biomarkers, but there is no relevant research in CRC. In contrast with Liu and colleagues, we chose the human protein interaction network as the background network for our study, which can reflect the biological functions of the individuals as a whole and has more biological significance. Second, as did Liu and colleagues, we all applied the three basic rules of the DNB method, but the research purpose and research methods were different. The transition point of cancer found by Liu and colleagues fell on a specific clinical stage, while we focused more on genes contributing to the disease transition, which will facilitate the detection of targeted drugs in the future. In addition, before using the DNB method to find DNBs, we explored the mutation sequences of genes in patients during cancer development, created mutation propagation modules based on the mutation sequences of genes, and then detected the critical transition points before disease.

In this study, we used multiomics data of CRC to obtain a specific mutant giant cluster (GC) featuring the identified mutant genes by a network propagation method. The biological significance of GCs and a high degree of consistency between GC and cancer-related pathways were confirmed by functional enrichment analysis of GCs. Subsequently, we used mutation selection rules to determine the mutation sequences of the mutant genes and used a dynamic network method to mine each patient’s specific DNBs. These DNBs can identify the transition point from normal to cancer in tumor progression. Clinical data verification showed that the transition point we obtained is in line with clinical staging. Due to the high tumor heterogeneity of CRC, we comprehensively considered the driver gene mutation sequences and DNBs of patients with the same type of disease and identified drug targets that could block the cancer process related to DNBs. This strategy provides important reference value for the diagnosis and treatment of CRC, especially for pre-emptive treatments, and is of great significance for reducing the incidence and mortality of CRC. Recently, there have been many studies identifying CRC biomarkers, but these studies have limitations in experimental data and experimental methods as well as in the accuracy, sensitivity and specificity of the identified biomarkers. In our study, each cancer sample had a tumor-adjacent sample compared with it. Under the premise of ensuring accuracy, the heterogeneity of CRC was considered. Using the concept of DNBs to explore the dynamic characteristics of CRC, we can better identify the early warning signals of sudden cancerous changes in the pre-disease state and achieve a true early cancer warning to prevent disease.

## Materials and Methods

### Data Collection

In this study, the expression profiles of 41 CRC patients were downloaded from The Cancer Genome Atlas (TCGA^1^). Each patient had a pair of cancer tissue and tumor-adjacent tissue expression data. The mutation profiles of 399 CRC patients were also obtained from TCGA. A total of 28 patients had both expression and mutation data, which were used in this study. Clinical information such as tumor staging and consensus molecular subtype (CMS) classification of these 28 patients was obtained from the cBioPortal website (Gao et al., 2013) and the CRC Subtype Consortium (CRCSC) website (Guinney et al., 2015). At the same time, 699 driver genes of CRC were obtained from DriverDB (Chung et al., 2016). A total of 39,240 experimentally confirmed human protein–protein interactions (PPI) were downloaded from the HPRD (Keshava Prasad et al., 2009). After preprocessing of the data, such as de-duplication, removal of isolated nodes, removal of self-interactions and loopback interactions, 36,867 pairs of 9,453 genes were used to construct a human PPI network for subsequent study.

**FIGURE 1:**
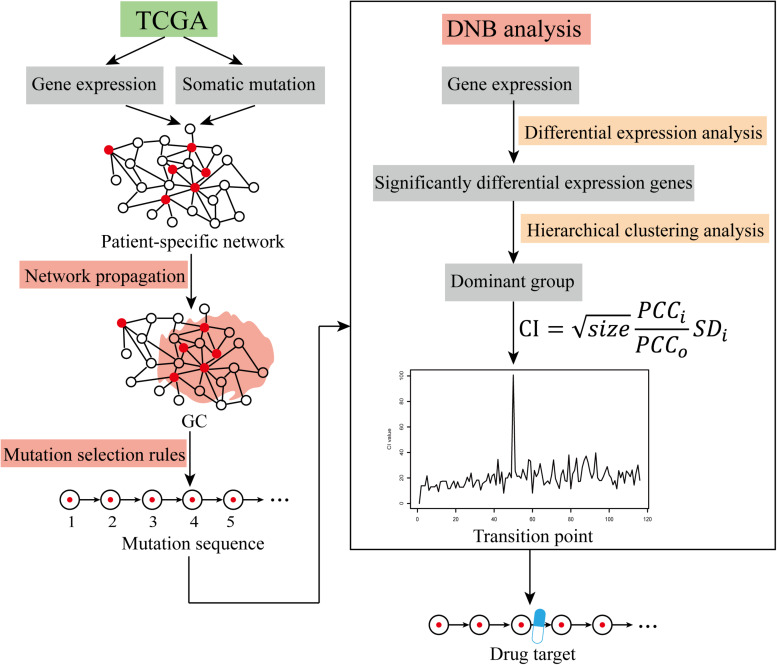
The workflow of cancer warning signal identification. Identification of cancer warning signals using TCGA data via the network propagation method, mutation selection rules, and DNB analysis.

The expression data and mutation data of each CRC patient were mapped into the PPI network to obtain a patient-specific network. The red nodes in the network represent mutant genes, and the white nodes represent non-mutated genes. Patient-specific GC mutations were obtained using network propagation analysis. In each patient, the mutation selection rule was applied to the GC to obtain the mutation sequences of driver genes. By this method, the mutated driver genes are sequenced as sequential changes during tumor development. Dynamic network analysis was used to find critical transition points through differential expression analysis, clustering analysis, and calculation of the criticality index (CI) to obtain patient-specific DNBs. Drug targets that block cancer progression were further explored in terms of their ability to recognize the DNBs (The workflow was shown in Figure 1). In the cluster analysis, we used the function pamk in the R package fpc for unsupervised clustering and selected the default optimal number of clusters. When searching for transition points, for the individual differences of patients, we only selected the maximum value of each patient’s CI and did not specifically focus on the range of change of each patient’s CI value.

### Construct the Patient-Specific Networks

For each patient with CRC, the expression profile was mapped to the PPI network. Since each patient’s expression profile had two expression datasets (one of the cancer tissues and one of the tumor-adjacent tissues), to ensure the specificity of the patient network, we selected gene pairs that interacted in the network with expression values for each gene in the two tissue samples not both being zero; in this way, the specific network of each patient was generated.

### Network Propagation of Mutation Effects

To simulate the propagation of mutation effects through the PPI network, we employed the network propagation method (Vanunu et al., 2010), which utilizes the random walk with restart (RWR) approach within a network. PPIs have often been screened at the proteome scale for many organisms, revealing 1000s of physical interactions between proteins (Valdeolivas et al., 2019). The availability of large-scale PPI networks led to the application of graph theory-based approaches for their exploration, with the ultimate goal of extracting the knowledge they contained about cellular functioning. These methods exploit the tendency of functionally related proteins to lie in the same network neighborhood (Schwikowski et al., 2000; Katsogiannou et al., 2014; Arroyo et al., 2015; Chapple et al., 2015).

Random walk with restart is the state-of-the-art guilt-by-association approach (Valdeolivas et al., 2019). A patient-specific network (undirected network) is defined as G = (V, E). The adjacency matrix of the network is defined as A, and M denotes a transition matrix that is the column normalization of A. An imaginary particle starts a random walk at an initial node v_0_∈V. Considering that the time is discrete, t∈N, at the t-th step, the particle is at node v_t_. Then, it walks from v_t_ to v_t+__1_, a randomly selected neighbor of v_t_ following matrix M. In the RWR version, at each iteration, the particle can also restart by jumping to any randomly selected node in the graph with a defined restart probability, r∈(0,1). This prevents the walk from being trapped in a dead end. Moreover, we can restrict the restart of the particle to a specific seed, setting each patient’s mutant gene as the seed. In doing so, the particle will explore the graph focusing on the neighborhood of the seed and measure the proximity between the seed and all the other nodes in the graph. Then, the RWR equation can be defined as follow:

(1) $$P_{t+1}^{T}=\left(1-r\right){\mathrm{MP}}_{t}^{T}+{\mathrm{rP}}_{0}^{T}$$

The vector P_0_ is the initial probability distribution. Therefore, in P_0_, only the seed has values different from zero. Each random walk only has one seed node, which is a mutant gene. Random walks are performed for as many mutations as the current patient has. After several iterations, the difference between the vectors P_t+__1_ and P_t_ becomes negligible, the stationary state is reached, and the elements in these vectors represent a proximity measure from every graph node to the seed. In this work, iterations are repeated until the difference between P_t_ and P_t+__1_ falls below 10^–10^ (Li and Patra, 2010; Erten et al., 2011; Zhao et al., 2015). We set the global restart parameter to *r* = 0.7 (Kohler et al., 2008; Li and Li, 2012; Smedley et al., 2014).

The above method results in a series of mutation propagation modules centered on the mutant seed gene in each patient. The mutation propagation modules overlap each other to form interconnected clusters, and we maximize the connectivity in the interconnected clusters in the patient-specific network. The cluster is called the mutant GC. This GC contains a maximum of the number of mutant genes and the mutation propagation module formed by the patient, which can fully reflect the state of mutation of the patient and has certain biological significance.

### Mutation Selection Rules

The development of cancer is accompanied by the gradual accumulation of somatic mutations. In the process of somatic mutation, finding the sequence of mutations in time is crucial for blocking the malignant development of cancer. It also has value for guiding research and understanding the occurrence and development of tumors. In the development of tumors, somatic mutations follow certain mutation selection rules. Based on the existing research results (Shin et al., 2017), we employed the mutation selection rule to select the next mutated gene that minimized the size of the connected module between a mutation candidate and all the previously mutated genes among mutation candidates overlapping with any previously mutated ones. The rule describes two opposing drivers of cancer development, one of which is to reflect the process of promoting cancer progression by ensuring an intersection with the current mutation propagation module and the other of which is to reflect the inhibition of cancer progression by minimizing connective clusters. These two driving forces simulate the confrontation process between cancer development and the immune system in the human body. The specific process is as follows:

Step 1: We selected an initial mutation among all the mutations except driver mutations.

Step 2: According to the rule, we determined the next mutation to be added at each evolution time step.

Step 3: We repeated the previous steps starting from all the available initial mutations and finally obtained as many mutation sequences as the order of the total number of somatic mutations of the patient.

Step 4: By investigating the order of a pair of mutations in the resulting mutation sequences across patients, we constructed a matrix that exhibits the number of mutation sequences such that one mutation in a row occurs earlier than the other mutation in a column.

Step 5: From the mutated gene sequence matrix, the order of mutations of any mutation gene pair can be obtained, and then the mutation sequence of all mutation genes in the patient GC is obtained.

### Dynamic Network Biomarker (DNB) Analysis

To identify predictive biomarkers for early diagnosis and prevention, and to understand the mechanism of disease development, we introduce DNB method. These DNBs can detect early warning signals that warn of sudden deterioration before the critical transition occurs with only a small number of samples. As Supplementary Figures S1, S2 show, as mutations accumulate, at one specific point, DNBs will be expressed differently from other genes and exert a significant influence on the following processes. Based on non-linear dynamic theory and the measured data, we theoretically and numerically showed (Chen et al., 2012; Li et al., 2017) that if there is a dominant group of molecules or genes satisfying the following three criteria from the observed data, then the system is near the critical state or tipping point, and the dominant group contains the DNBs we are looking for:

**FIGURE 2:**
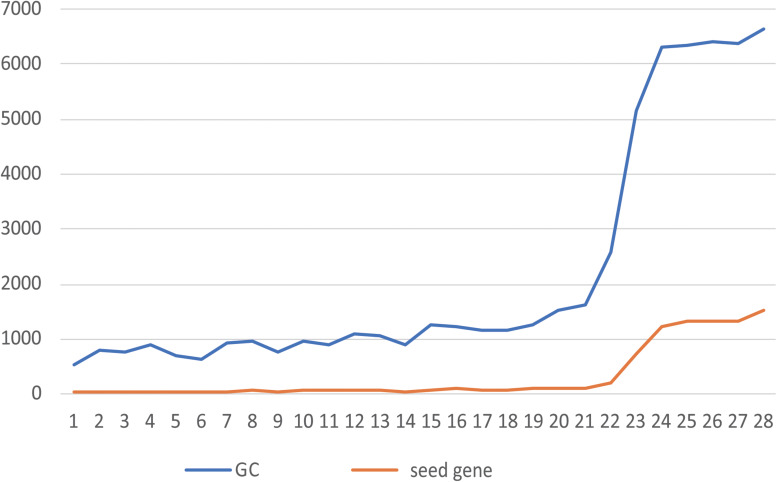
Relationship between the number of seeds and the size of the GC. The abscissa shows the NO. of each of 28 patients, and the ordinate shows the number of genes. The blue line is the number of genes contained in the GC, and the orange line is the number of seeds contained in the GC.

(1) Each member of the dominant group fluctuates violently;
(2) The correlation between any pair of members in the dominant group becomes very strong;
(3) The correlation between members of the dominant group and other non-dominant group members becomes very weak.

The following quantification index (CI: criticality index) approximately considering all three criteria can be used as the numerical signal of the DNB method:

(2) $$CI=\sqrt{\mathrm{size}}\frac{{\mathrm{PCC}}_{i}}{{\mathrm{PCC}}_{o}}{\mathrm{SD}}_{i}$$

where *size* is the number of molecules in the dominant group or DNB, SD_i_ is the average standard deviation of all molecules in the dominant group, PCC_i_ is the average PCC of all molecule-pairs in the dominant group (absolute value), and PCC_*o*_ is the average PCC of molecule-pairs between the dominant group and others (absolute value). That is, from the measured data, the appearance of a group of genes (or proteins) with strongly collective fluctuations indicates an imminent critical transition (i.e., the system is near the critical state). Thus, the group members are the predictive/dynamic biomarkers for this critical (generally irreversible) transition. When the CI reaches a peak or increases markedly during the measured periods, the biological system is at the critical period or tipping point (Yang et al., 2018). The DNB distinguishes not only normal samples from disease samples but also pre-disease samples from disease samples using both molecular fluctuation information (i.e., dynamic information) and network information (i.e., correlation information among molecules), in contrast to traditional static biomarkers.

## Results

### Basic Information of Patients

In this study, data from patients with CRC, including cancer sample expression data, tumor-adjacent sample expression data, and mutation data, were extracting. A total of 28 patients, 3 patients had clinical stage 1 tumors, 14 patients had clinical stage 2 tumors, 5 patients had clinical stage 3 tumors, and 5 patients had clinical stage 4 tumors, and the other 1 patient had no clinical staging information. According to the CRCSC, 4 patients had CMS1, 8 patients had CMS2, 3 patients had CMS3, and 9 patients had CMS4, and the other 4 patients had no CMS typing information. In addition to CMS typing information, we also analyzed microsatellite instability/microsatellite stability (MSI/MSS) typing information for the CRC patients. Details of the 28 patients with CRC can be found in Supplementary Table S1.

### Patient-Specific Networks

Based on the expression data from the cancer sample and the control sample of each CRC patient, a specific network for each patient was constructed. The genes in the network were expressed in at least one of the cancer samples and the control sample. The node is completely dependent on the patient’s own gene expression and has strong specificity, providing an accurate and highly personalized basis for subsequent research. The basic information of the patient-specific networks is shown in Supplementary Table S1. From the statistical results, we found that the number of genes in each patient’s specific network and the number of interactions between genes were very similar; however, the number of mutations in each patient was significantly different. These networks fully reflect the differences between patients and reflect the necessity of personalized treatment. By visualizing the patient-specific networks, we found that the mutated genes and the non-mutated genes were closely associated in the network, and there were one-to-many and many-to-many interaction relationships. Therefore, in each patient, studying a certain mutant gene alone would not reflect the overall situation of the patient. The mutation function module consisting of mutant genes and non-mutant genes was selected as the research object. We mined the mutation propagation module in the patient-specific network to explore the mutation information of the patient.

### Mutation Propagation Module

Using the network propagation method, the mutation genes of each CRC patient were used as the seeds, and the patient-specific network was used as the background network to obtain many mutation propagation modules centered on the mutant seeds. As one seed migrates through the network, its own mutational effect will decrease as the number of walking steps increases. That is, the closer the gene is to the seed, the higher influence the seed will have, and the higher the degree of correlation with the seed is, the higher the final score will be; on the contrary, nodes with low scores are not included in the mutation propagation module composed of the mutant genes. To ensure high correlation between each node in the mutation propagation module and the seed gene, we compared multiple thresholds (0.0001, 0.0005, 0.001, and 0.005). The score threshold for mutation propagation was finally determined to be 0.001, which means that the nodes affected by the seed genes with a score greater than 0.001 were retained to form a mutation propagation module. This threshold value also satisfies that the size (number of genes) of the minimum mutation propagation module is not zero under the premise of ensuring high correlations between each node in the mutation propagation module and the seed genes. Among the mutation propagation modules of all patients, the size of the largest propagation module was 205, and the size of the smallest mutation propagation module was 1. The module size is shown in Supplementary Table S1.

### Giant Clusters (GCs)

When all the seeds of the patient form mutation propagation modules, which are scattered in the patient-specific network, some genes are shared by multiple propagation modules. Multiple modules are connected to each other to form a module cluster by sharing the same gene. The module cluster is a subnetwork formed by two or more mutant seeds. We choose the largest connected module cluster, called the giant cluster (GC). The GC is the largest subnet formed by the interconnection of mutation propagation modules dominated by patient mutated genes that may have a significant impact on patient-specific networks. The relationship between the number of seeds and their corresponding GC size for 28 patients is shown in Figure 2.

In Figure 2, the greater the number of seeds, the more genes are contained in the GC. By counting the proportion of genes contained in the GCs of 28 patients in their specific networks, it was found that from the 23rd patient (the 24th patient begins Figure 2), when the number of seeds was greater than 1,000, the proportion of GCs in the network increased from 10% to more than 70% (Supplementary Table S1). This indicates that the accumulation of mutant genes in CRC patients leads to changes in the number and function of genes affected by GC from quantitative to qualitative, which is consistent with the tumor development process. By calculating the proportion of seeds in the GC to all seeds, we can conclude that the GC contains more than 90% of the mutant seeds in the patient (Supplementary Table S1). It is indicated that the information contained in the GC is sufficient to reflect the patient’s mutation state. A subsequent study of the GCs confirmed our conjecture.

**FIGURE 3:**
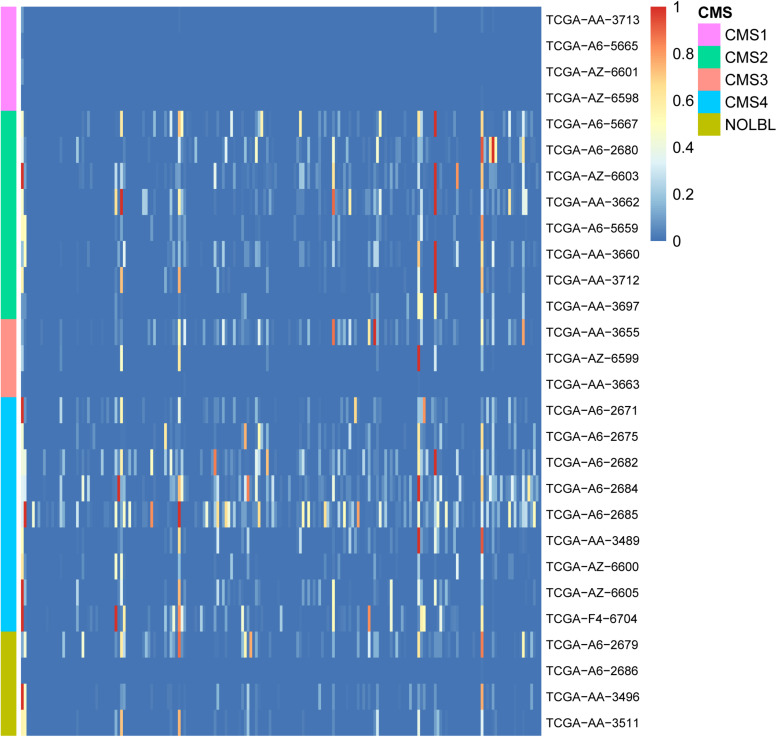
Heatmap of enrichment analyses of 28 patient GCs in 189 cancer-related pathways. The horizontal axis shows 189 cancer-related gene sets, and the vertical axis shows the GCs of 28 patients. The color gradient in the figure from red to blue represents the corrected *P*-value of the hypergeometric test. The smaller the *P*-value, the more significantly the genes in the GC are enriched in the cancer-related gene set.

To study the biological processes involved in GC and the relationship with cancer in patients with CRC, 189 cancer-related gene sets were downloaded from the Molecular Signature Database (MSigDB; Subramanian et al., 2005; Liberzon et al., 2011). Functional enrichment analysis was performed on the GCs of 28 patients with 189 cancer-related gene sets. We believe that the GC contains enough information on patient mutations. Therefore, if the genes in the GC are widely enriched in cancer-related pathways, they play a significant role in the development of tumors. The enrichment results are shown in Figure 3. In the enrichment heatmap, as seen from the large blue color in the figure, the genes contained in GC are highly correlated with cancer, participate in cancer-related biological processes, and play a significant role in the occurrence and development of tumors. In addition, as shown in the coordinates on the left side of the figure, the enrichment analysis results can be used to cluster different CRC patients with CMS. It can be seen from the figure that the heat maps of patients with different CMS types are very different. By the Wilcox rank sum test, the *P*-value between every two types was less than 0.05, so the difference was significant. The biological functions of GC in patients with different CMS classifications are inconsistent. Therefore, as shown in the figure, GC can distinguish different types of patients well.

Through various comparisons and functional analyses, GC has a high correlation with CRC patients. The seed and non-seed genes in GC can represent cancer occurrence, development, CMS typing, etc. Therefore, it is meaningful to detect mutation sequences in GC.

### Mutation Sequence of Mutant Genes in GC

As the GC can represent the modified biological function of CRC patients, the mutation sequences of the mutant genes in the GC can be found to describe the occurrence and development of tumors in different patients. However, in the process of tumor development, there are 100s of genetic mutations in tumor tissues, but most of them are passenger mutations that represent neutral mutations (Zhang and Wang, 2019). Only a small portion of them will actually cause cancer and play a key role in tumor development. These mutations are driver mutations. According to the set mutation rules, the mutation sequences of the driver genes in the GC represent the development of the tumor. The mutation sequence can be used to define the time at which the tumor state changes and further to find the critical transition point at which the tissue transforms from normal to cancer in the process of cancer development.

Since the mutated genes of each CRC patient are different, the resulting driver gene mutation sequences are also different (Supplementary Table S2). To determine a clinically relevant mutation sequence, we analyzed the mutation order of samples from patients with the same CMS with the mutation selection rule, taking the heterogeneity of CRC into account, so that patients with the same CMS will show a uniform mutation sequence (Supplementary Table S3). We believe that this integrated sequence can be used clinically as a reference for judging the progression of cancer in CRC patients.

### DNB Analysis

We used the mutation sequence of the driver genes in each patient’s GC as the standard. The dynamic network method has been applied. The change of the CI (criticality index) reflects the changes of gene expression and biological function during the development of the disease. By counting the increase of the CI in different periods of each patient, it was found that the CI will reach a peak or a significant increase within a certain period. During this period, the genes of the dominant group showed strong collective fluctuations. We believe that this period is the critical point of transition. At the critical transition point in each patient, the timely use of drugs can prevent CRC occurrence and development.

**FIGURE 4:**
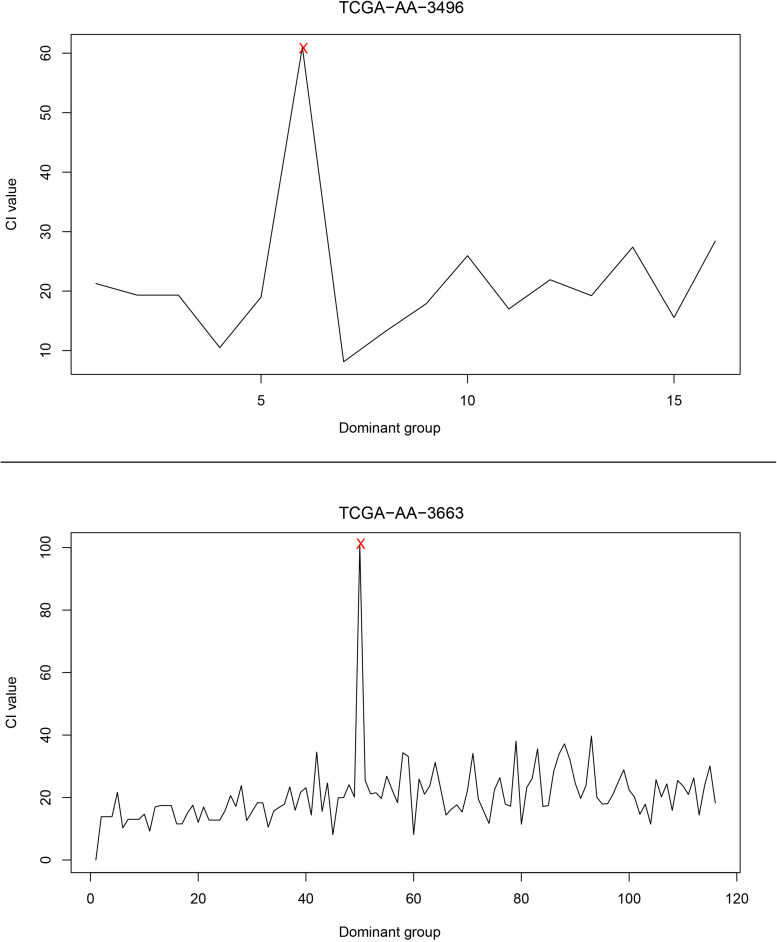
The curve of the CI. The upper part of the figure is the CI curve of the TCGA-AA-3496 sample, and the lower part is the CI curve of the TCGA-AA-3663 sample. The horizontal axis shows the dominant group dominated by the driver genes in each patient’s GC, and the vertical axis shows the CI of the corresponding dominant group.

Next, the relationship between CI changes and the number of driver genes in each patient was examined. Figure 4 shows changes in the CI of the TCGA-AA-3496 (16 driver genes in the GC) and TCGA-AA-3663 (116 drivers in the GC) samples. Regardless of the number of driver genes, a period in which the CI reaches a peak (marked in red) can be found in each patient. After that point, the CI returns to a relatively stable fluctuation state. We believe that this period is the turning point of the patient’s cancer, in which the dominant group genes are the DNBs of the corresponding patient. See the Supplementary Figure S3 for the CI curves for all the patients.

### Verification of the Clinical Applicability of the Transition Point

To validate the clinical applicability of the transition points based on CRC typing, we grouped patients by different clinical stages. In patients with the same CMS, we determined whether the driver genes involved in the transition point of patients in early disease stages were mutated before those of patients in advanced disease stages. Taking CMS1 patients as an example, there were 4 patients with CMS1, 3 who were in clinical stage 2, and 1 who was in clinical stage 4. By analyzing the mutation order of each patient and the corresponding transition point, we found that the driver genes that were mutated before the transition point (EP300) of patients in stage 4 included a transition point gene (SPERT) of patients in stage 2. However, in the mutation sequence of the patients in stage 2, the transition point genes of the patients in stage 4 were not included, except for in one patient in whom the transition point gene was EP300. Therefore, for patients with CMS1, the order of mutations at the transition point is consistent with the chronological sequence of clinical stage. That is, the stage 2 transition point genes were mutated prior to the stage 4 transition point genes. In addition to the clinical staging validation results in patients with CMS1, the same validation results were obtained in other subtypes (Table 1). This finding confirms the clinical applicability of the transition point genes we found. It also demonstrates the credibility of our mutation sequences and the accuracy of potential anticancer targets.

**TABLE 1:** Clinical staging verification for driver transition points in patients with the same CMS.

	Stage 1	Stage 2	Stage 3	Stage 4
CMS1		SPERT/AR		EP300
CMS2	COL6A3			AR
CMS2		LRP1B/FAT4/ANK2	MAP1B/NFASC/LRP2/SCN3A/LPA	
CMS2			LRP2	AR
CMS3	PCLO/CREBBP	CACNA1S/EGFR		
CMS4		CTNNB1		SMAD4
CMS4		CTNNB1	PTEN/NFASC/RUNX1T1	

### Finding Drug Targets That Block Cancer Progression

The mutation sequences in driver genes found in CRC patients can be used to find drug targets that can block cancer process. The dominant groups of genes at the transition point for patients of the same CMS were integrated, called DNB_CMS_i_, where i is a different type of CMS, and i = 1, 2, 3, 4; in addition, we integrated the driver genes at the transition point with the driver genes at the previous point of the transition point, called Driver_CMS_i_. Similarly, i is a different type of CMS, and i = 1, 2, 3, 4. After integrating the data, the relevant functional pathways were identified in the KEGG database, and the pathways needed to meet the following conditions:

(1) A pathway must containing at least two driver genes in the Driver_CMS_i_ gene set;
(2) The anteroposterior regulation sequence of at least two driver genes in the pathway must be the same as the sequence of mutations after integration;
(3) Between the two driver genes in condition (2), there are genes that are not in the Driver_CMS_i_ gene set but are in the DNB_CMS_i_ gene set. These genes represent candidate drug target genes.

**FIGURE 5:**
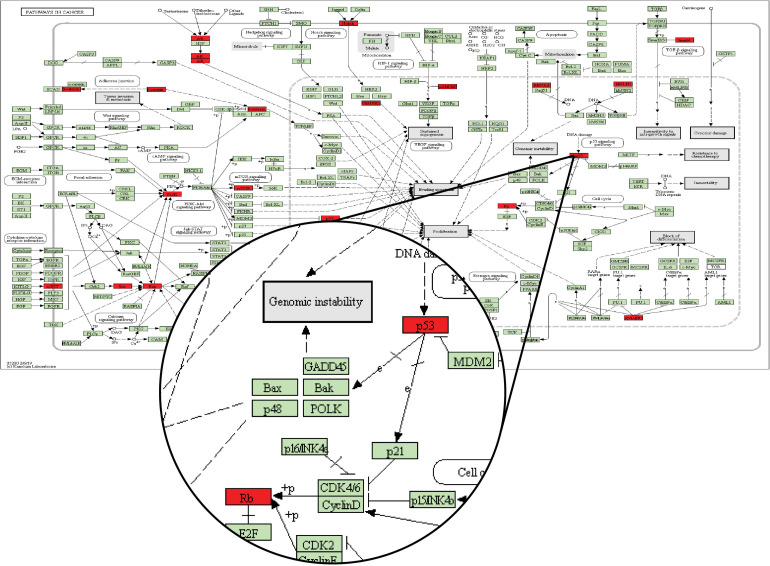
KEGG database pathway hsa05200: pathways in cancer. The green node is the default gene of the pathway map, and the red node is the driver gene of the Driver_CMS_i_ gene set.

Through a manual search, CMS1 samples included eight candidate drug target genes, namely, *AKT1, CALM1, CCND1, CCNE1, CDK2, MDM2, PXN*, and *RELA*. These genes frequently appeared in the KEGG pathways identified for two driver genes in the Driver_CMS_1_ gene set. For example, the candidate drug target *CCND1* is located between the two driver genes in the pathways hsa05200: pathways in cancer, hsa05220: chronic myeloid leukemia, hsa05214: glioma, and hsa05218: melanoma. The sequence of the two driver genes was consistent with the order of mutations we found (Figure 5).

In the pathway map of Figure 5, *p53* corresponds to the driver gene *TP53*, and *Rb* corresponds to the driver gene *RB1*. From the pathway, we can see that the position of *TP53* in the pathway is before *RB1*. In the mutation sequence in our integrated CMS1 patients, *TP53* is also mutated prior to *RB1*. Moreover, *CCND1* is the gene encoding Cyclin D and is found between *TP53* and *RB1* in the pathway is in the DNB_CMS_1_ gene set of the transition point dominant group of patients with CMS1. Therefore, *CCND1* is a candidate drug target for blocking the progression of cancer in CMS1 patients.

Similarly, CMS2 included four candidate drug target genes, named *PLCG1, PIK3R1, VAV1*, and *VAV3*; CMS3 included six candidate drug target genes, named *PLCG1, MDM2, CALM3, MAP2K1, MAPK1*, and *PLD1*; and a total of two candidate drug target genes were found in CMS4, named *TGFB1* and *SMAD3*. See Supplementary Table S4 for details.

In each type, there are specific candidate drug targets with high specificity. These drug targets were frequently identified in patients with the same CMS but not in patients with other subtypes. For CRC, a cancer with high tumor heterogeneity, different drugs can be used to target subtype-specific genes, which could improve the therapeutic efficacy and reduce side effects. However, at the same time, many aspects need to be considered before a protein can be used as a drug target, such as molecular weight, polarity, and tissue distribution in the body (Hopkins and Groom, 2002; Bakheet and Doig, 2009). Therefore, we further studied the existing drug target information and candidate drug targets to explore adaptive anticancer drug targets.

We used the existing drug-target interaction data and our 18 candidate drug target genes to extract specific anticancer targets and drugs, which might be used in cancer treatment. After we annotated the 18 candidate drug target genes with the drug-target information, 12 known drug targets (Table 2) were identified, of which 6 had been used as anticancer drug targets in clinical treatment. We hope that using these six drug targets can block the development of specific cancers in time and achieve the goal of pre-emptive treatment. In our opinion, the six identified targets, which are closely related to the occurrence and progression of cancer, including anti-inflammatory targets and nutritional factors, will have the potential to become anticancer drug targets and will be used in anticancer drug repositioning.

**TABLE 2:** Potential anti-colorectal cancer targets in existing drug targets.

Gene	Target	Target type
AKT1	P31749	Anticancer drug target
CALM1	P0DP23	Anticancer drug target
CCND1	P24385	Anticancer drug target
CDK2	P24941	Anticancer drug target
MAP2K1	Q02750	Anticancer drug target
MAPK1	P28482	Anticancer drug target
PIK3R1	P27986	Anti-inflammatory target
TGFB1	P01137	Anti-inflammatory target
MDM2	Q00987	Nutrition-related target
CALM3	P0DP25	Nutrition-related target
PLD1	Q13393	Nutrition-related target
RELA	Q04206	Other

## Discussion

This study constructed a patient-specific network with patient expression data. The genes in this network were determined by patient-specific expression data. We know that the expression profiles of patients with different tumor stages and different cancer subtypes are different. Due to tumor heterogeneity, the expression profiles of patients with the same tumor stage and the same cancer subtype are not the same. Therefore, we obtained each patient-specific network based on this theory (a typical network map can be found in Supplementary Figures S1, S2). Such networks will aid the development of individualized treatments and provide more accurate medical guidance for patients with the same tumor stage and same cancer subtype. Through network propagation analysis, CRC-specific mutant GCs were obtained. The GCs covered more than 90% of the patients’ mutation information. These clusters were highly correlated with cancer-related gene sets and provide a good reference for studying mutations in CRC. The mutation selection rule was used to obtain the mutation sequence of driver genes in CRC patients. The driver gene mutation sequences of patients with the same CMS were considered simultaneously. This analysis not only considered the tumor heterogeneity of CRC but also provides a reference for the diagnosis of the clinical CRC stages. Finally, we used dynamic network analysis to mine DNBs in CRC patients. These DNBs were able to identify the dynamic progression of the tumors, representing a critical transition point between normal to cancer and during cancer progression from one stage to another. The results obtained were verified by clinical data and mirrored actual clinical staging. At the same time, the biological pathways identified by KEGG analyses were further utilized to exploit drug targeting DNBs that can block the progression of cancer. These results provide important value for the diagnosis and treatment of CRC, especially for pre-emptive treatment. It is of great significance to reduce the incidence and mortality of CRC.

At present, there are many research results identifying CRC biomarkers. For example, Shin et al. (2017) studied the mutation sequence of five key driver genes during the development of CRC, but no control samples were included in the study. Therefore, the study could only describe the development of tumors after cancer had appeared. In our study, each cancer sample had a tumor-adjacent sample. Therefore, the critical transition point from normal to cancer in the development of CRC could be found, which provides a reference for monitoring high-risk groups of patients with CRC. Another example is that Linda JW Bosch et al. (2017) identified the hypermethylation status of decoy receptor 1 (*DCR1*) as a biomarker for predicting metastatic CRC, but the accuracy and applicability were slightly lower. In our study, while ensuring accuracy, we also considered the heterogeneity of CRC. All patients with CRC were classified according to molecular subtypes, and a single and comprehensive analysis was performed for each patient. In the gene function analysis of patients with GC, it was found that the GCs of patients with the same CMS had a high degree of consistency, and there was a large difference between patients with different CMSs (*P* < 0.05). This also fully illustrates the necessity of typing analysis for CRC patients.

In recent years, many studies have found that for many complex diseases, the progress of the disease is not necessarily smooth but abrupt (Richard et al., 2016; Lesterhuis et al., 2017; Li et al., 2017). This transformation is the pre-disease state of disease progression. At this stage, if properly treated, the disease can usually be reversed back to normal, which means that the pre-disease state is an unstable state (Achiron et al., 2010). However, most of the current methods for finding CRC biomarkers focus on molecular (Chabanais et al., 2018) and network methods (Shin et al., 2017). They are static and are mainly used to distinguish between disease samples and normal samples, and it is difficult to identify pre-disease samples. Therefore, markers identified from such strategies lack the ability to diagnose disease early and interfere with the occurrence of disease. In our research, the concept of DNBs was employed. This dynamic network method was developed on the basis of non-linear dynamics and complex network theory (Chen et al., 2012; Liu et al., 2012). We used it to reflect changes in expression in cancer samples and control samples in the same patient to reflect changes in biological function in patients during CRC, which allowed us to explore the dynamic characteristics of CRC. This strategy can better identify the early warning signs of sudden cancerous changes in the pre-disease state and achieve a true early warning to prevent disease. The recurrence rate of patients after CRC surgery is a serious complication, considered as a failure of the therapeutic strategy (Duineveld et al., 2016; Farhat et al., 2019), and the changes in tissues adjacent to cancer are more susceptible. Therefore, our research can provide early disease diagnosis methods for patients with a family history of CRC or patients after CRC surgery.

Currently, it is common to apply network methods to the research and develop drugs for human diseases. We mapped the expression and mutation profiles of CRC patients to interaction networks with human protein interaction network data, creating a CRC-specific network so that we could reveal the dynamic changes in human patients in specific networks during the development of CRC. In recent years, network physiology has been a good method to study human diseases from a holistic perspective. The human body is an integrated network in which complex physiological systems, each with its own regulatory mechanisms, continuously interact and in which failure of one system can trigger a breakdown of the entire network (Bashan et al., 2012; Bartsch et al., 2015; Liu et al., 2015). The central task of statistical physics is to understand macroscopic phenomena that result from microscopic interactions among many individual components often driven by competing forces and non-linear feedback mechanisms (Ivanov and Bartsch, 2014). This type of analysis is also applicable to the complex mechanisms in physiology. The interdisciplinary field of network physiology bridges two active fields of modern science: (A) the physics of complex networks and (B) the organization and control of integrated physiologic organ systems (Ivanov et al., 2016; Moorman et al., 2016). Network physiology can identify and quantify the dynamic changes in humans during the development of diseases. Since our research objects are genes and many genes are expressed differently in various tissues and organs, there will be varying degrees of influence in different tissues and organs. Our results can provide a basis for the study of CRC from the view of network physiology.

When selecting experimental samples for such analyses, patient samples are required to have both cancer tissue expression data and corresponding tumor-adjacent tissue expression data, as well as mutation data of the patients. Therefore, the number of eligible samples in this study was small, only 28, and it was impossible to comprehensively study the genetic mutations and mutation sequences of CRC patients with different stages. At the same time, we classified the samples according to different molecular subtypes and obtained specific results. However, the result will be affected by the insufficient number of experimental samples, which might be a shortcoming of this research. We hope to add more experimental data in follow-up work to improve the results and provide a further comprehensive analysis.

## Data Availability Statement

All datasets generated for this study are included in the article/Supplementary Material.

## Author Contributions

XC and LL conceived and designed the study. ZS, FX, and SR performed the experiments. ZS wrote the manuscript. LL and JL reviewed the manuscript. All of the authors read and approved the manuscript.

## Conflict of Interest

The authors declare that the research was conducted in the absence of any commercial or financial relationships that could be construed as a potential conflict of interest.

## Footnotes

1 https://www.cancer.gov/tcga

## Supplementary Material

FIGURE S1 Four states in the dynamic cancer processes of sample TCGA-AA-3496. The network was constructed from the protein–protein interaction (PPI) network of the patients. **(A)** The process begins with a normal state, **(B)** then mutations accumulate during the process. **(C)** The pre-disease state was detected when the expression of DNBs in sample TCGA-AA-3496 fluctuated strongly, and this moment is the DNB warning state. **(D)** After the DNB warning state, the number of mutations increases dramatically, and patients are defined as having a disease state.

FIGURE S2 Four states in the dynamic cancer processes of sample TCGA-AA-3663. The network was constructed from the PPI network of the patients. **(A)** The process begins with a normal state, **(B)** then mutations accumulate during the process. **(C)** The pre-disease state was detected when the expression of DNBs in sample TCGA-AA-3663 fluctuated strongly, and this moment is the DNB warning state. **(D)** After the DNB warning state, the number of mutations increases dramatically, and patients are defined as having a disease state.

FIGURE S3 The CI curves of all the patients. The horizontal axis shows the dominant group dominated by the driver genes in each patient’s GC, and the vertical axis shows the CI of the corresponding dominant group.

TABLE S1 Basic information of 28 CRC patients.

TABLE S2 The patient’s driver mutation sequence.

TABLE S3 The driver mutation sequence of patients with the same CMS.

TABLE S4 Candidate drug target genes of patients with the same CMS.
