# Supplementary figures and images for: Identification of Early Warning Signals at the Critical Transition Point of Colorectal Cancer Based on Dynamic Network Analysis
Source: Front Bioeng Biotechnol. 2020 May 29;8:530. doi: 10.3389/fbioe.2020.00530 (PMC7272579; doi:10.3389/fbioe.2020.00530)

A

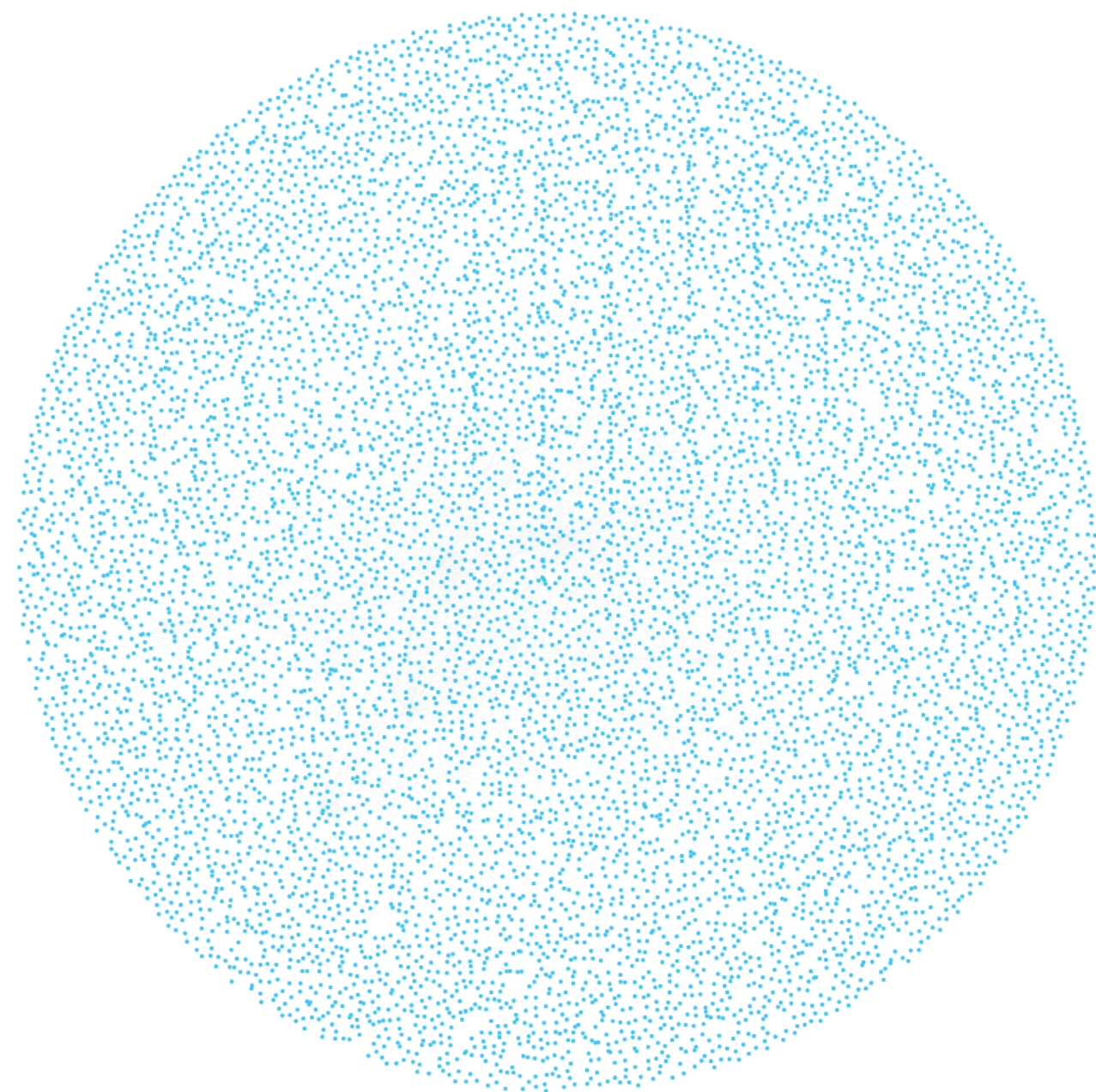

primary state

B

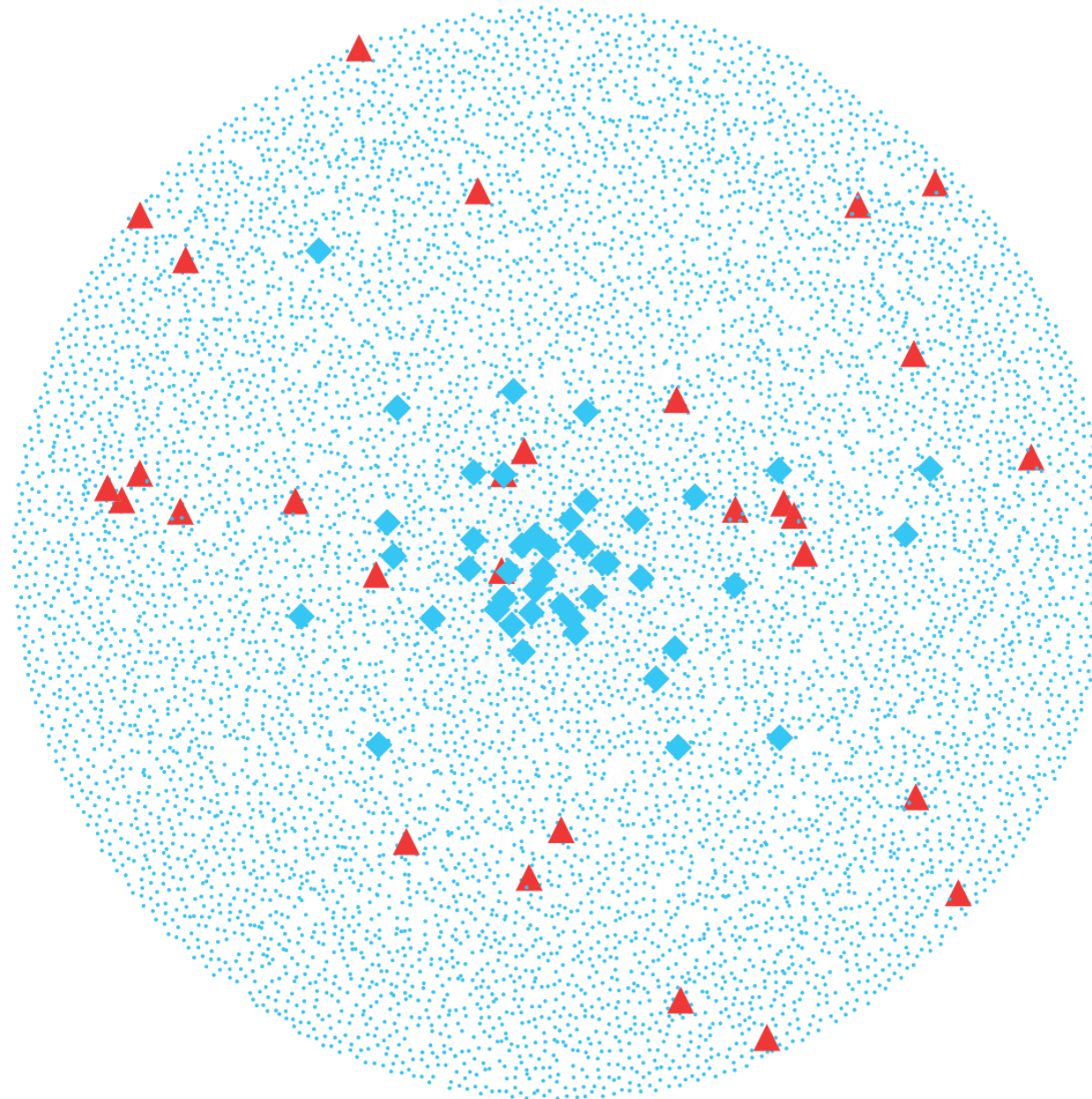

mutations accumulate

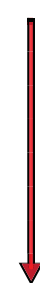

C

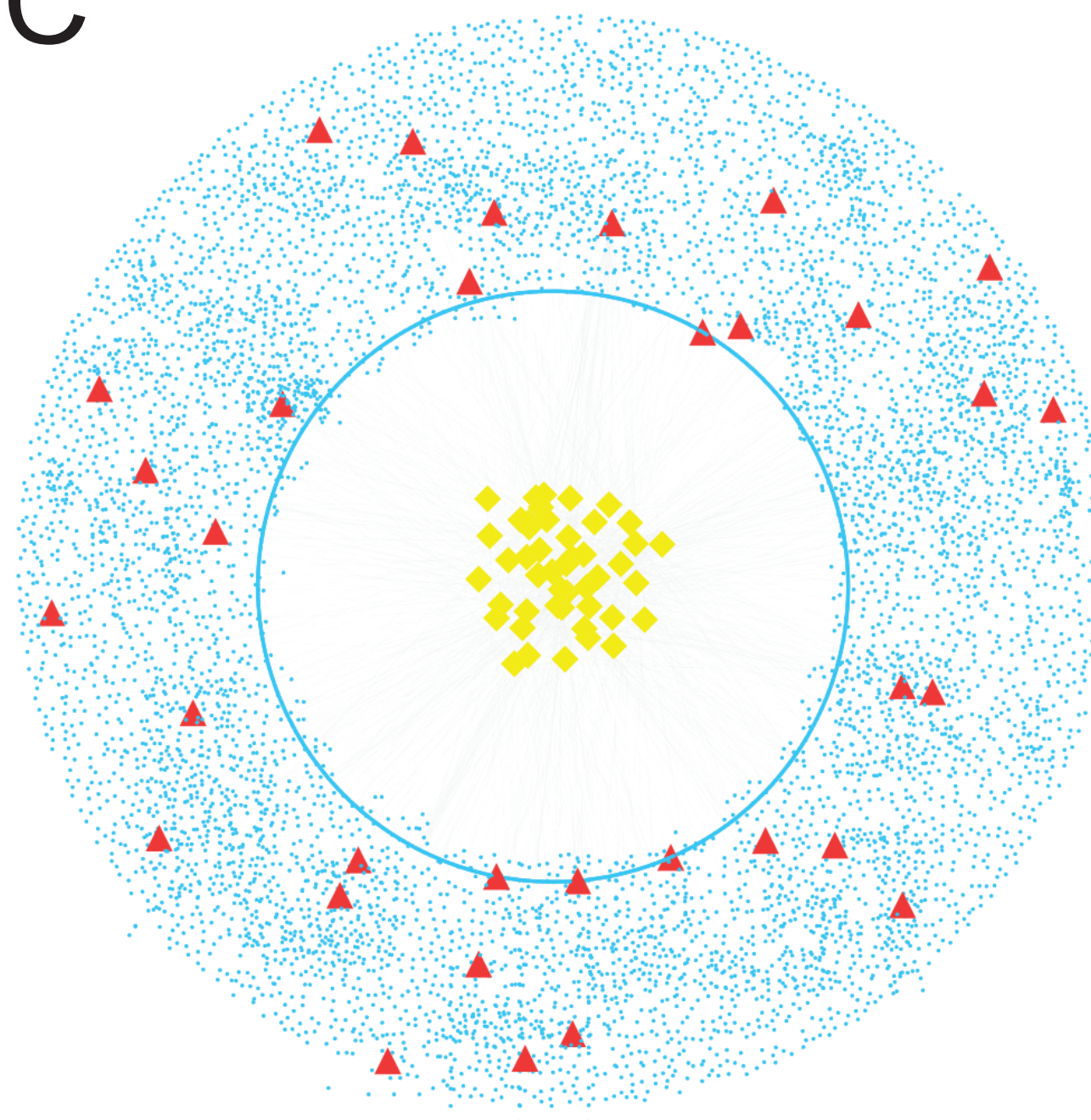

DNB warning

D

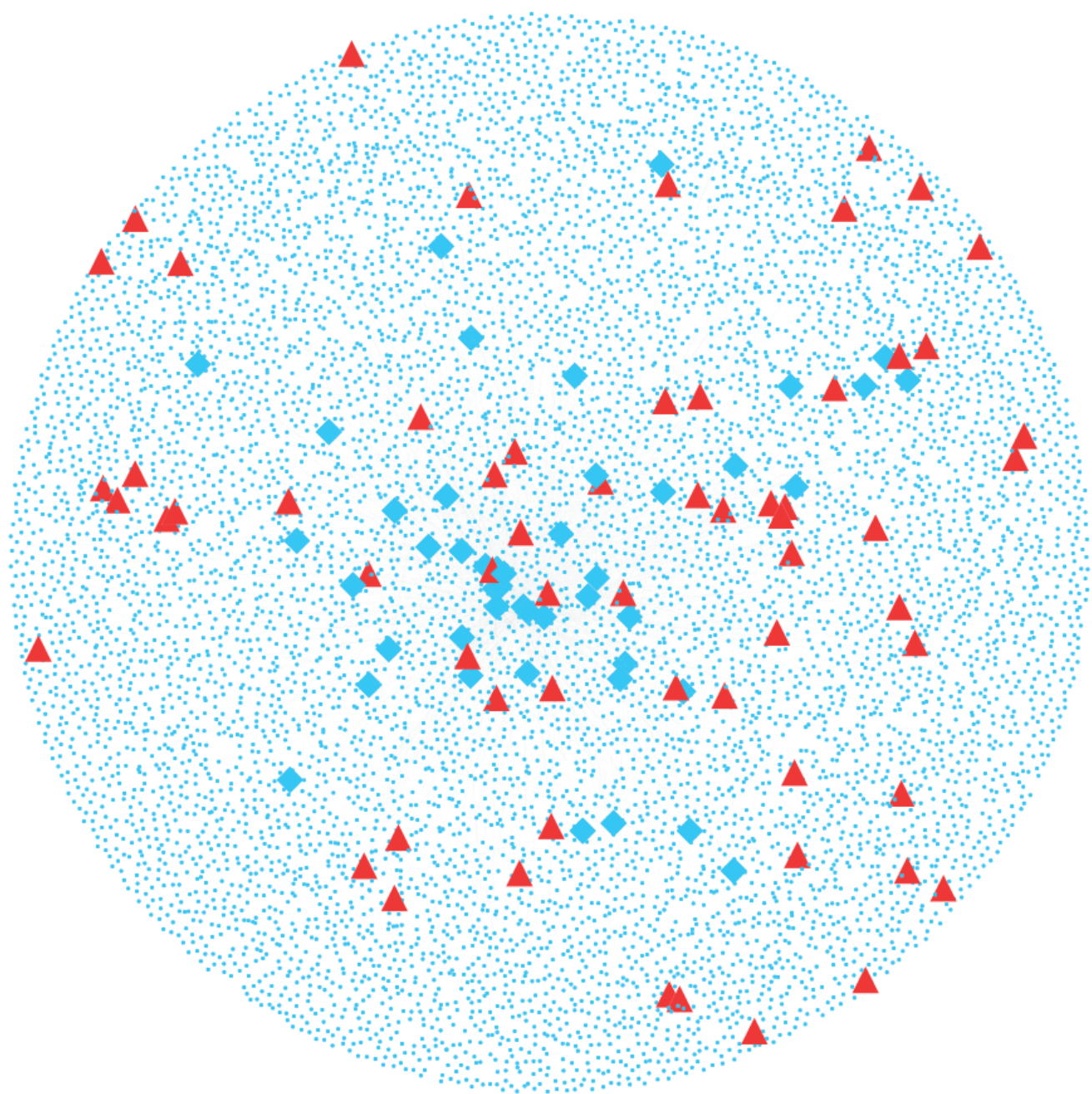

tumor state

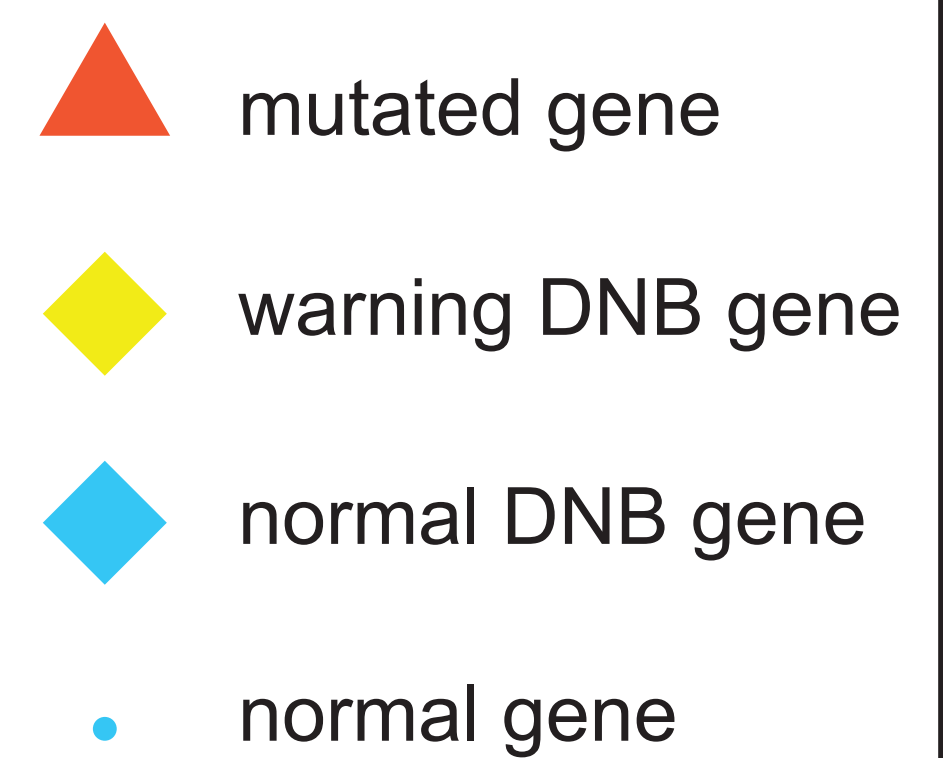

Supplement: FIGURE S1 — Four states in the dynamic cancer processes of sample TCGA-AA-3496. The network was constructed from the protein–protein interaction (PPI) network of the patients. (A) The process begins with a normal state, (B) then mutations accumulate during the process. (C) The pre-disease state was detected when the expression of DNBs in sample TCGA-AA-3496 fluctuated strongly, and this moment is the DNB warning state. (D) After the DNB warning state, the number of mutations increases dramatically, and patients are defined as having a disease state. [file Image_1.PDF]

A

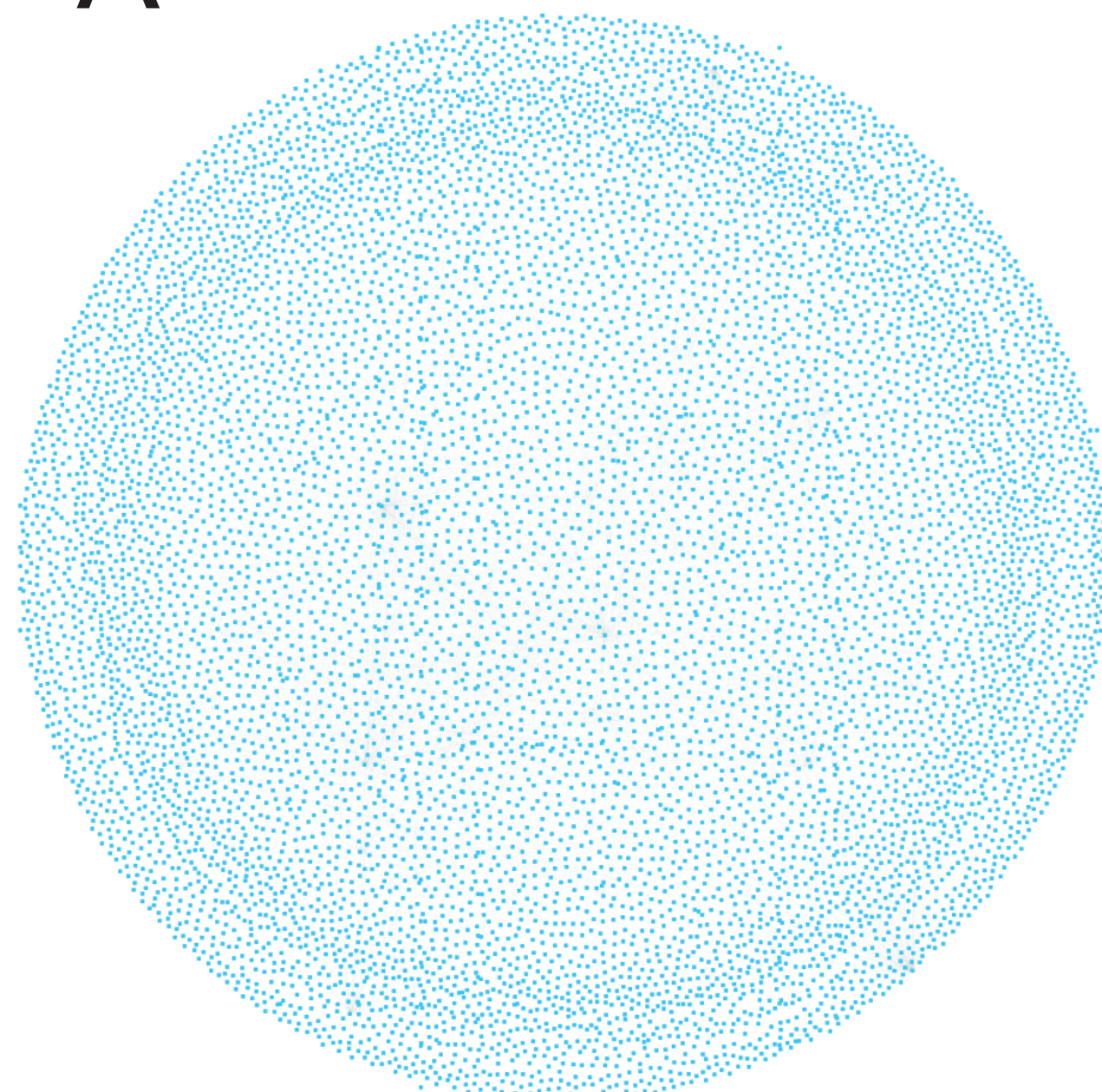

primary state

B

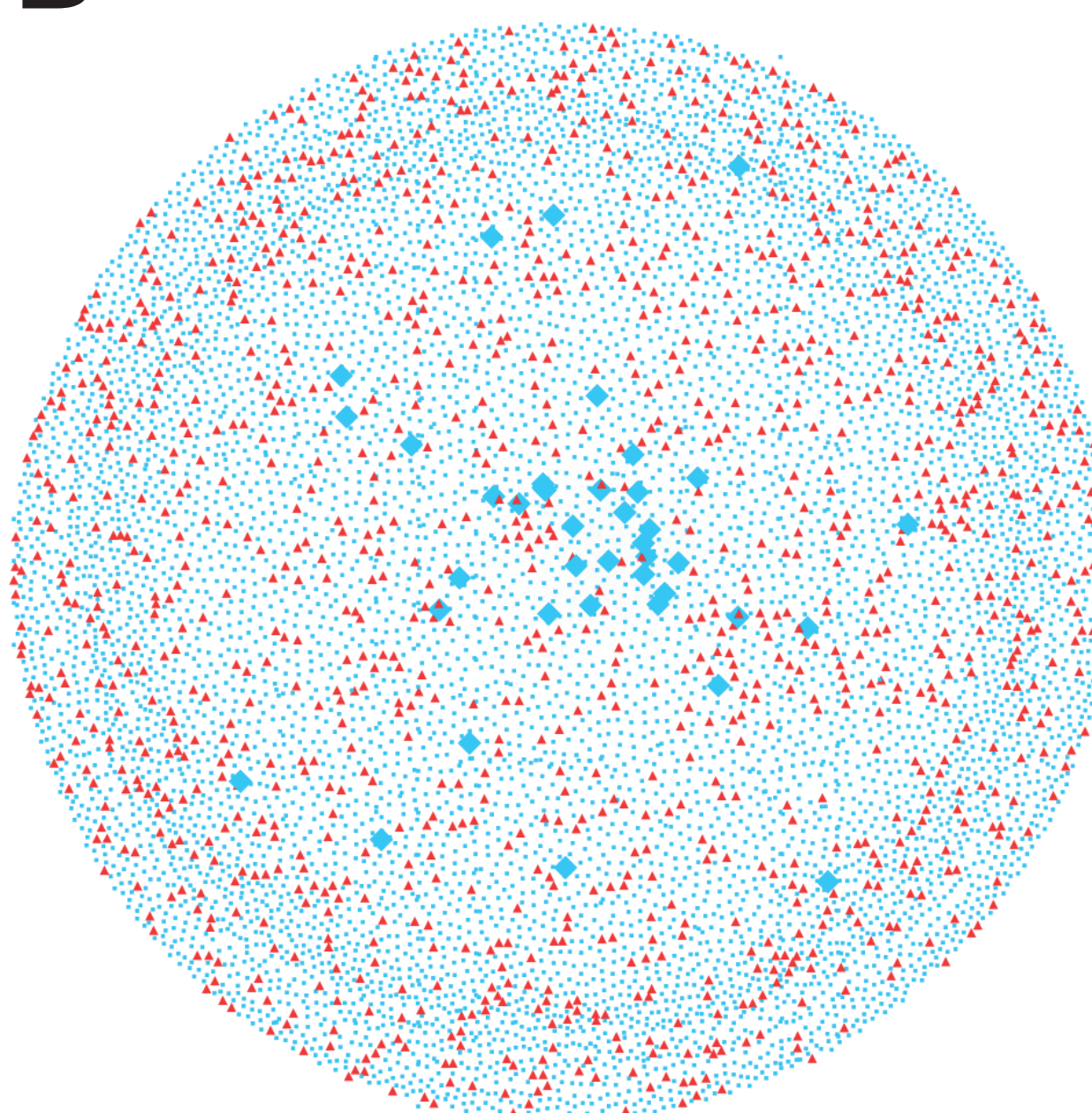

mutations accumulate

D

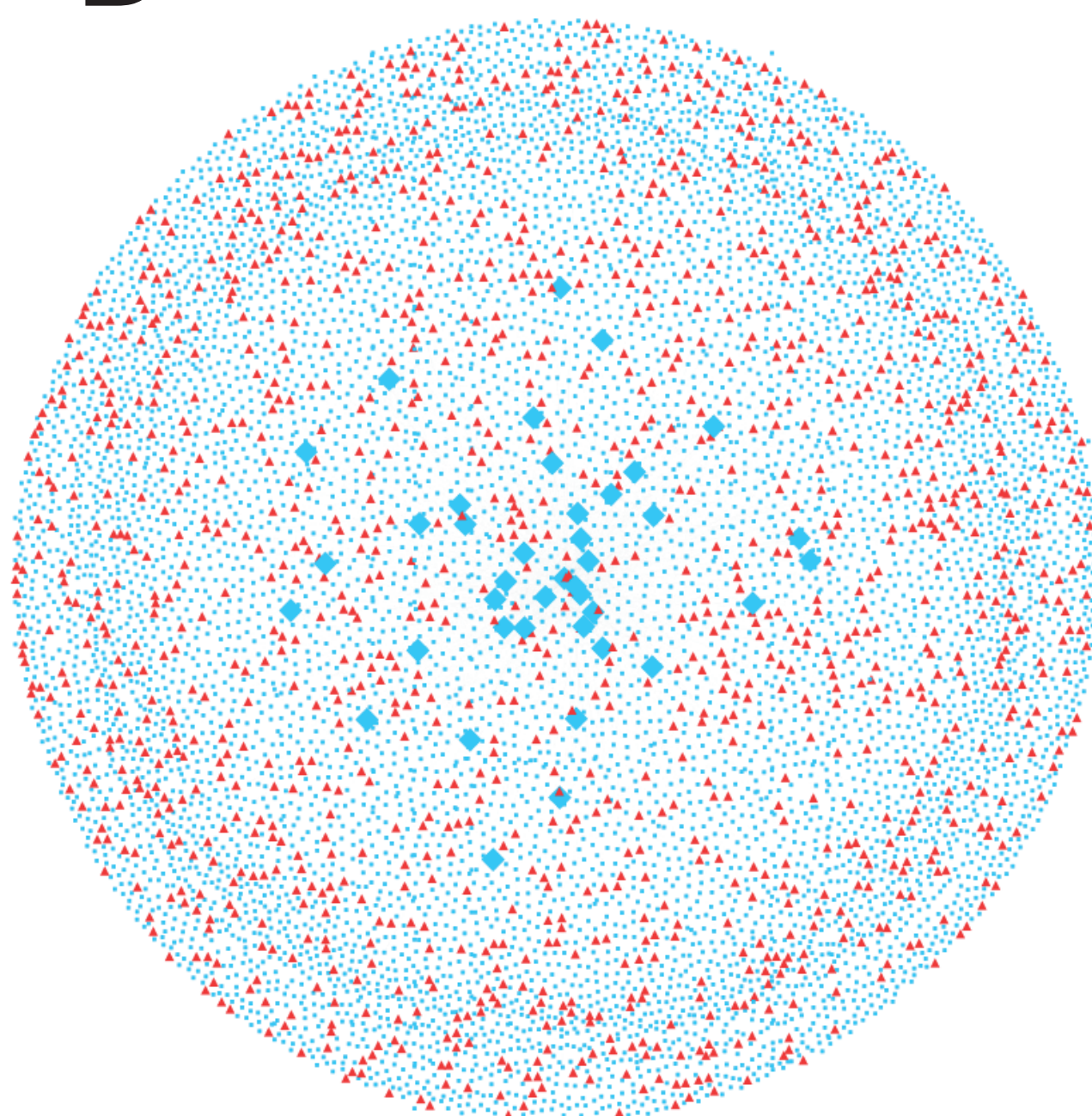

tumor state

C

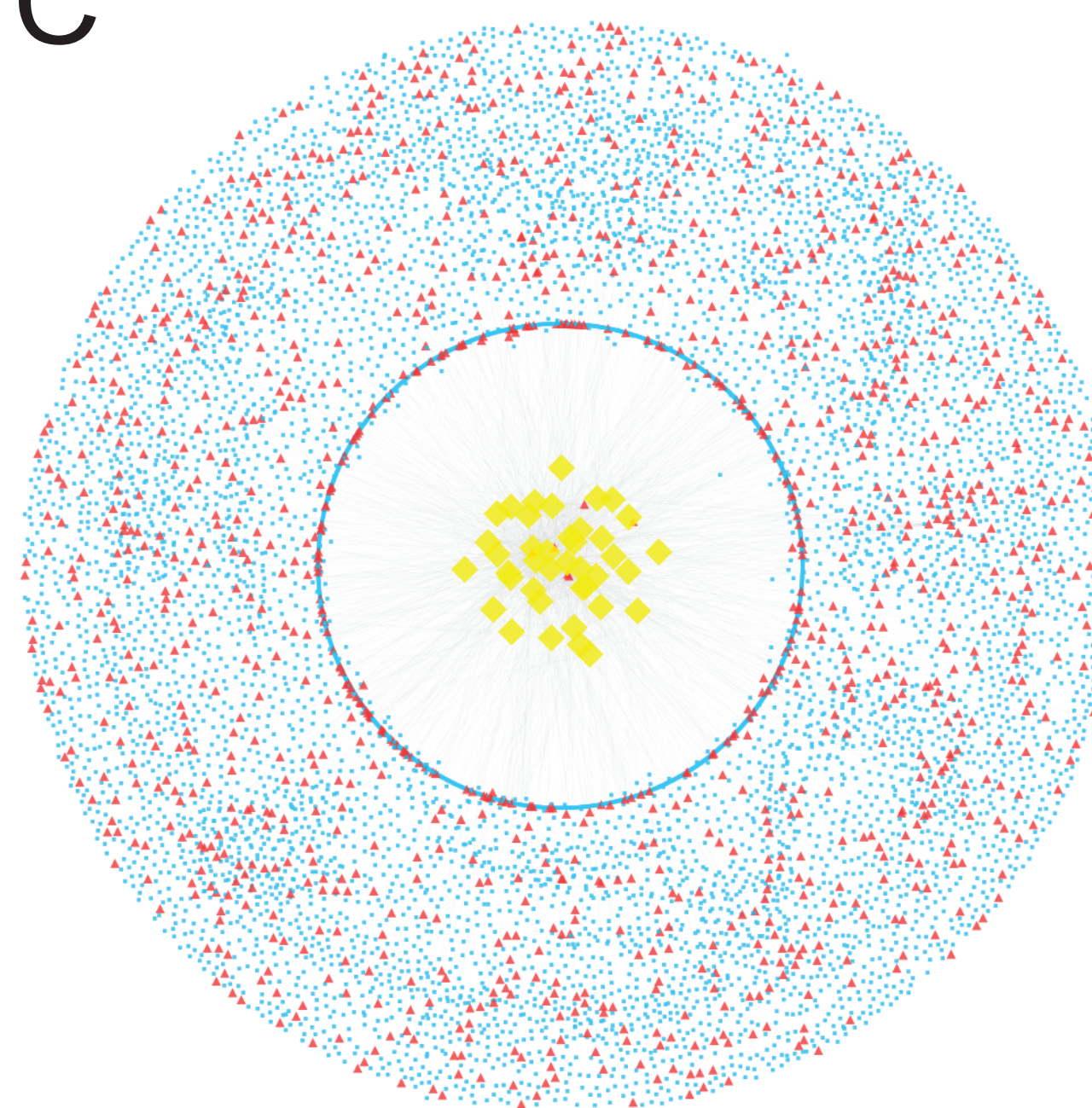

DNB warning

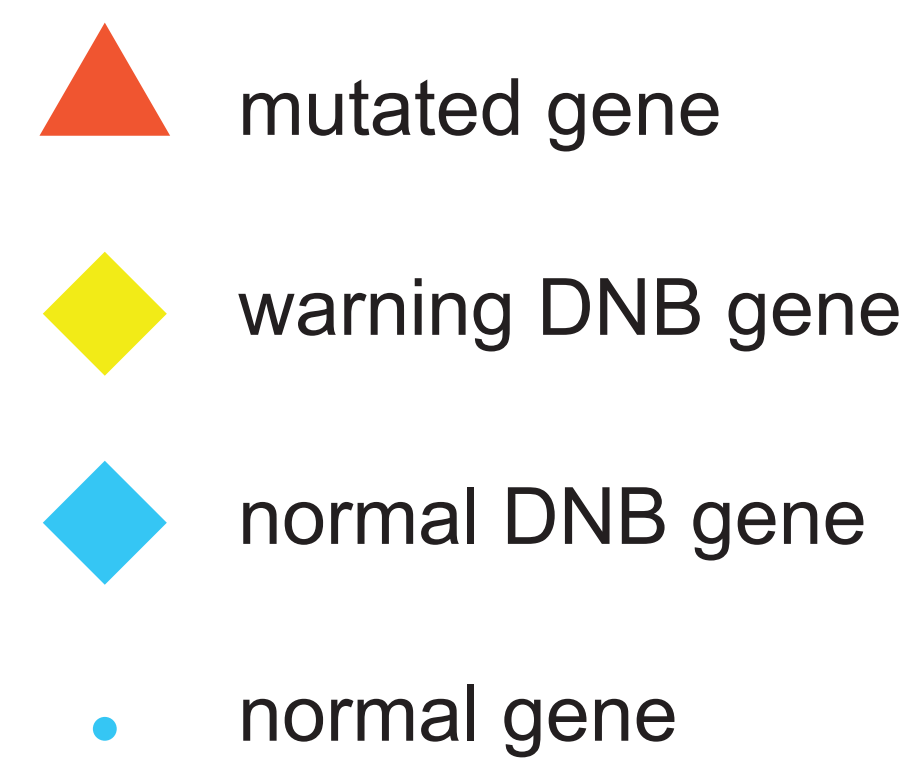

Supplement: FIGURE S2 — Four states in the dynamic cancer processes of sample TCGA-AA-3663. The network was constructed from the PPI network of the patients. (A) The process begins with a normal state, (B) then mutations accumulate during the process. (C) The pre-disease state was detected when the expression of DNBs in sample TCGA-AA-3663 fluctuated strongly, and this moment is the DNB warning state. (D) After the DNB warning state, the number of mutations increases dramatically, and patients are defined as having a disease state. [file Image_2.PDF]
