# Supplementary material for: Identification of Early Warning Signals at the Critical Transition Point of Colorectal Cancer Based on Dynamic Network Analysis
Source: Front Bioeng Biotechnol. 2020 May 29;8:530. doi: 10.3389/fbioe.2020.00530 (PMC7272579; doi:10.3389/fbioe.2020.00530)
Supplement: FIGURE S3 — The CI curves of all the patients. The horizontal axis shows the dominant group dominated by the driver genes in each patient’s GC, and the vertical axis shows the CI of the corresponding dominant group. [file Image_3.PDF]

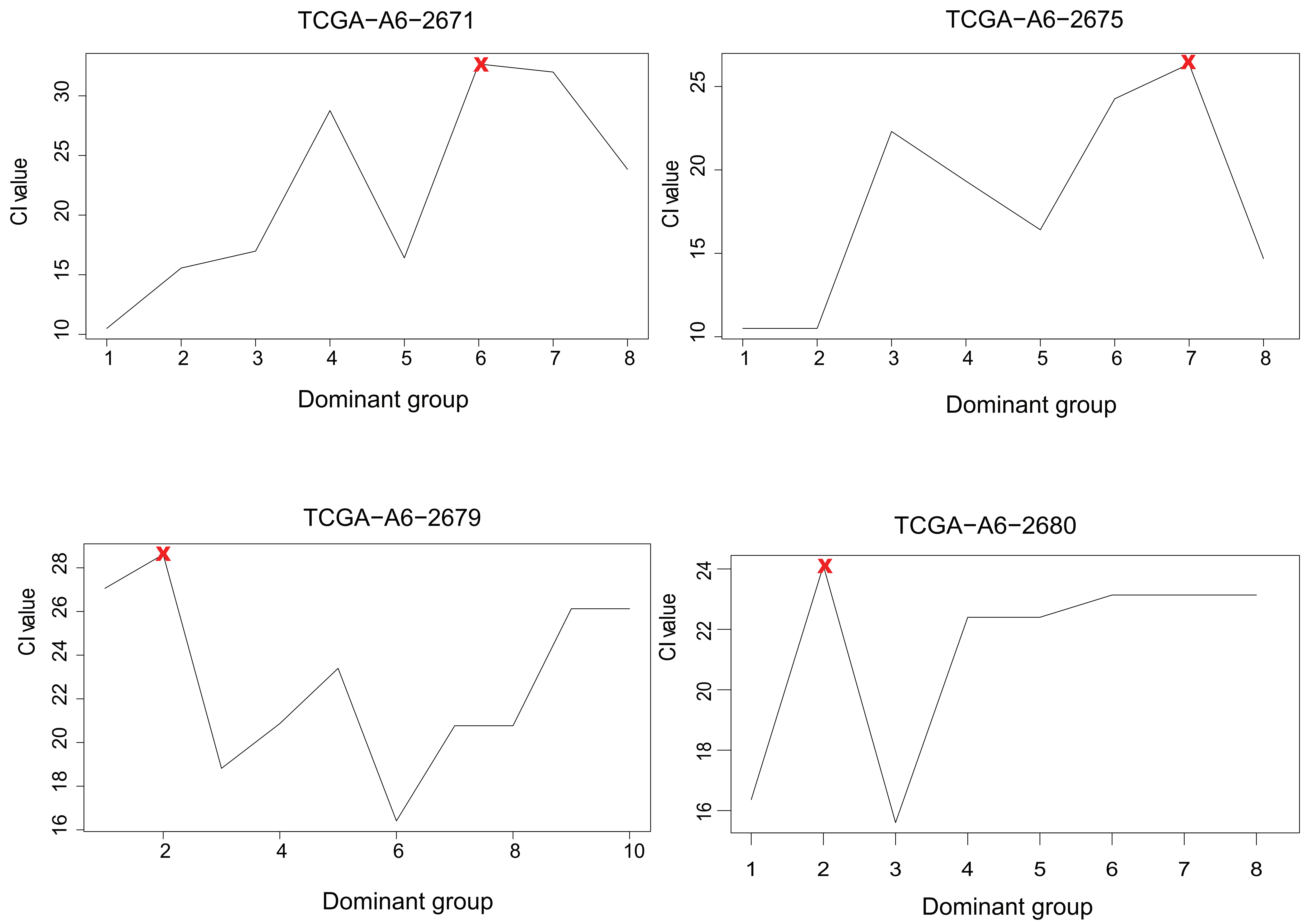
TCGA−A6−2671
TCGA−A6−2675
TCGA−A6−2679
TCGA−A6−2680
CI value
Dominant group

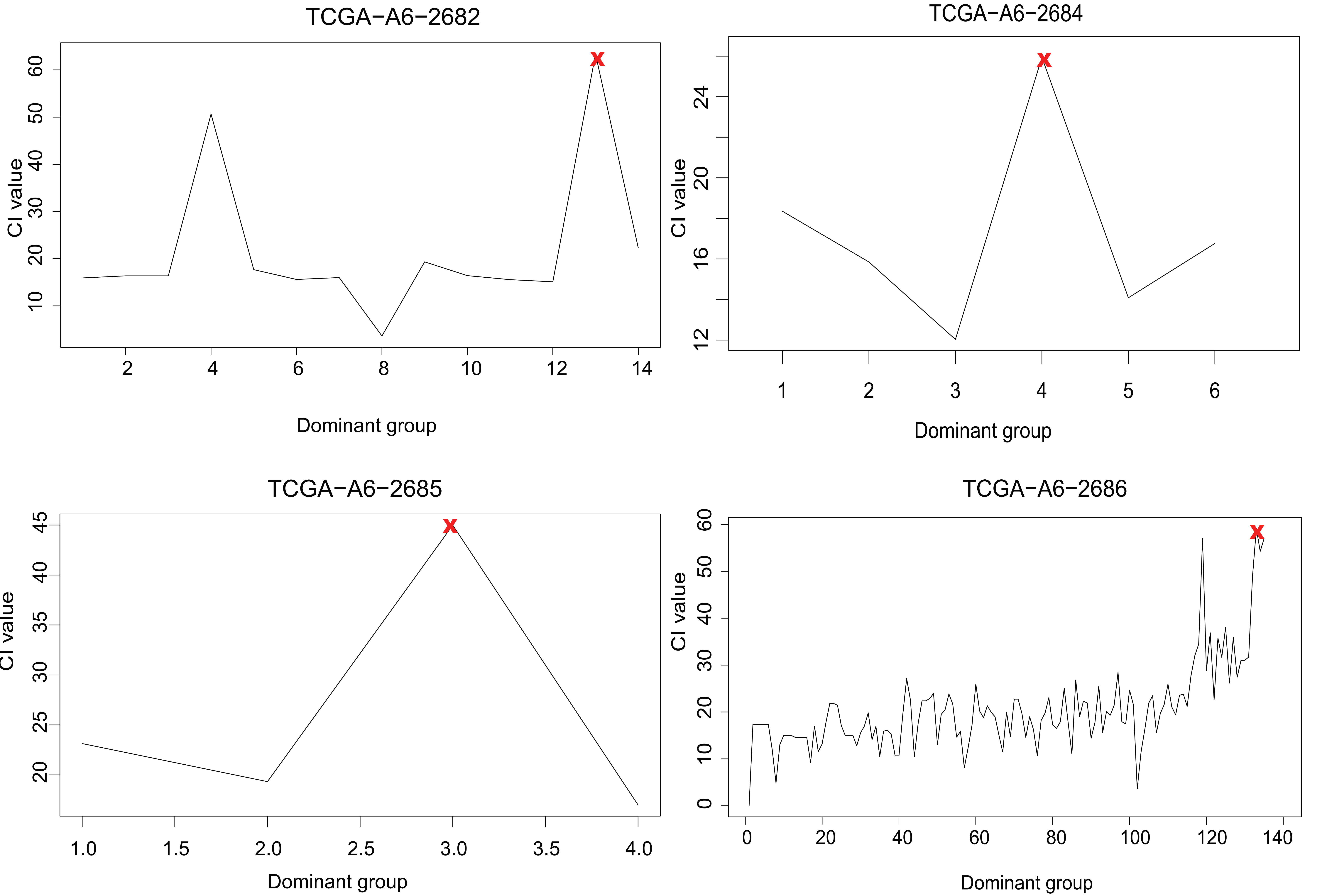
TCGA−A6−2682
CI value
Dominant group
TCGA−A6−2684
CI value
Dominant group
TCGA−A6−2685
CI value
Dominant group
TCGA−A6−2686
CI value
Dominant group

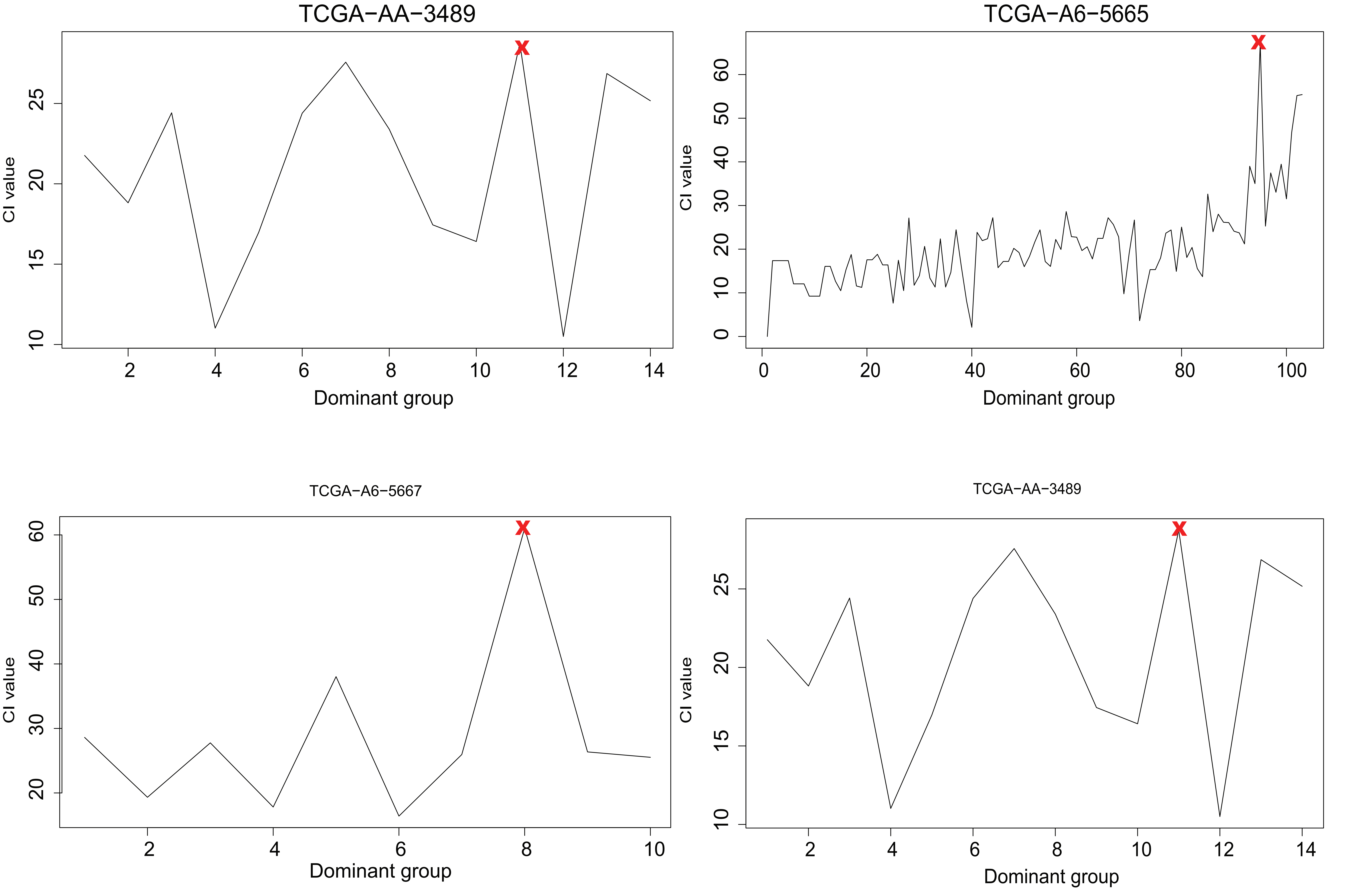

TCGA−AA−3489
CI value
10
15
20
25
2
4
6
8
10
12
14
Dominant group
X
TCGA−A6−5665
CI value
0
10
20
30
40
50
60
0
20
40
60
80
100
Dominant group
X
TCGA−A6−5667
CI value
20
30
40
50
60
2
4
6
8
10
Dominant group
X
TCGA−AA−3489
CI value
10
15
20
25
2
4
6
8
10
12
14
Dominant group
X

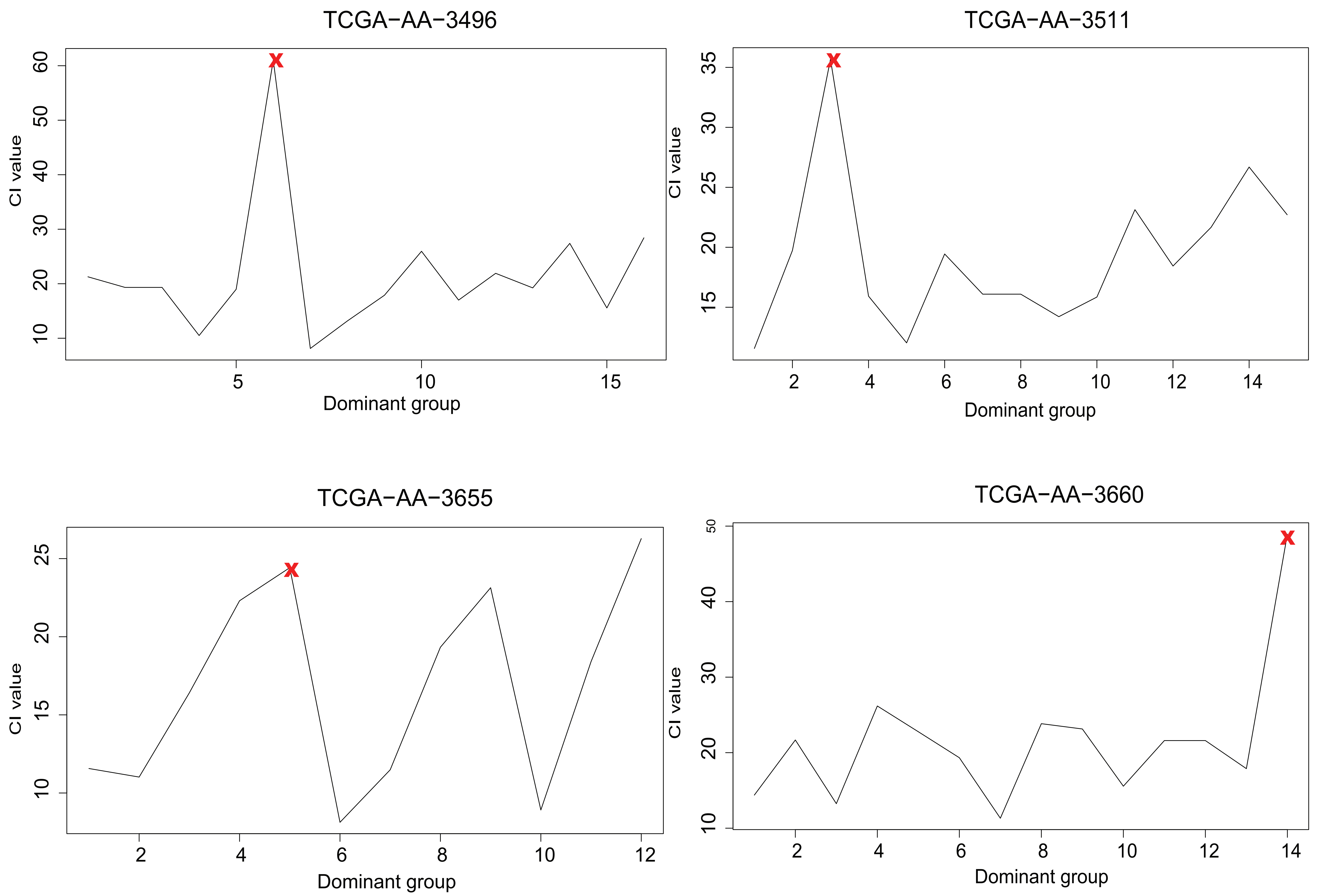

TCGA−AA−3496
CI value
10
20
30
40
50
60
5
10
15
Dominant group
TCGA−AA−3511
CI value
15
20
25
30
35
2
4
6
8
10
12
14
Dominant group
TCGA−AA−3655
CI value
10
15
20
25
2
4
6
8
10
12
Dominant group
TCGA−AA−3660
CI value
10
20
30
40
50
2
4
6
8
10
12
14
Dominant group

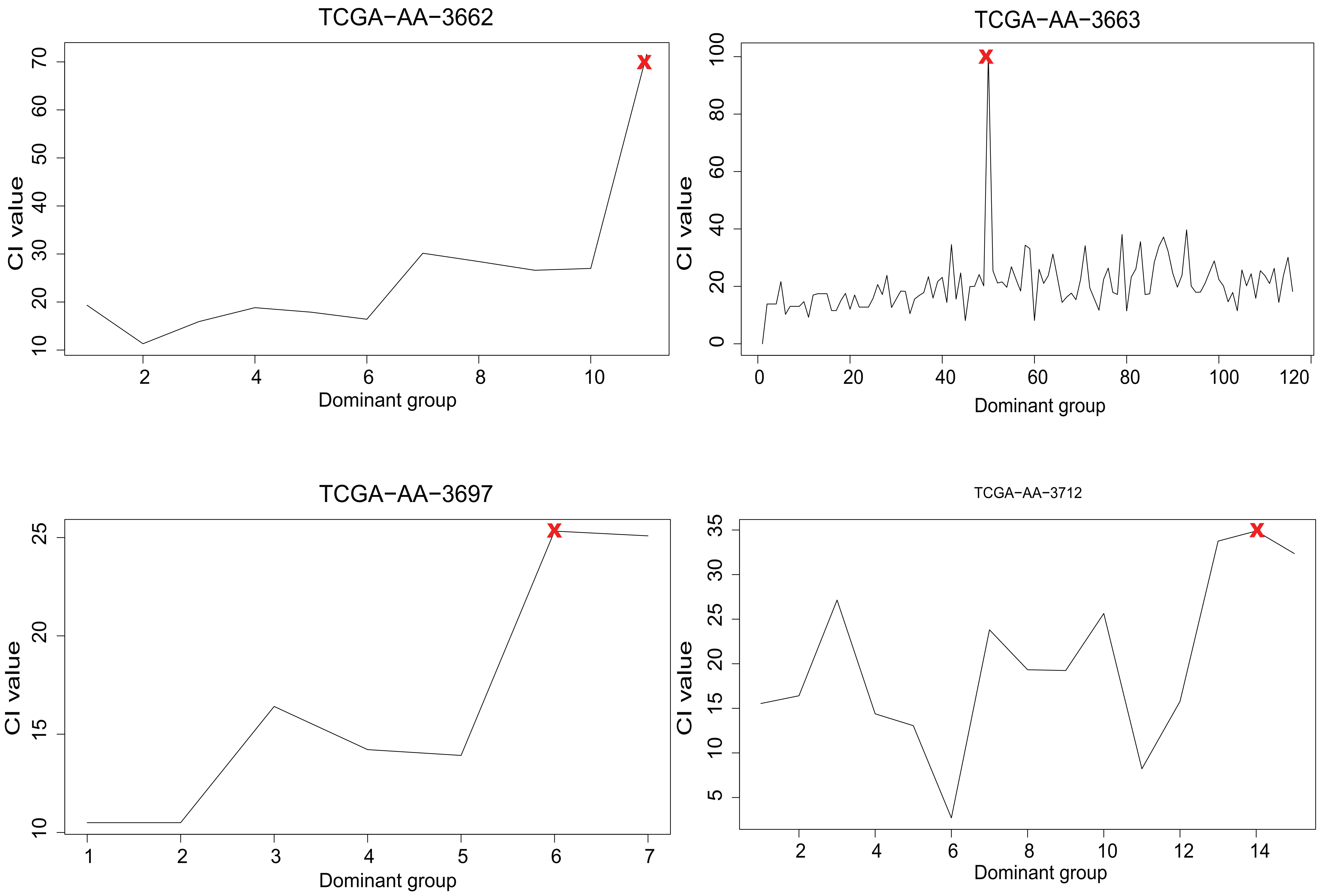
TCGA−AA−3662
CI value
Dominant group
TCGA−AA−3663
CI value
Dominant group
TCGA−AA−3697
CI value
Dominant group
TCGA−AA−3712
CI value
Dominant group

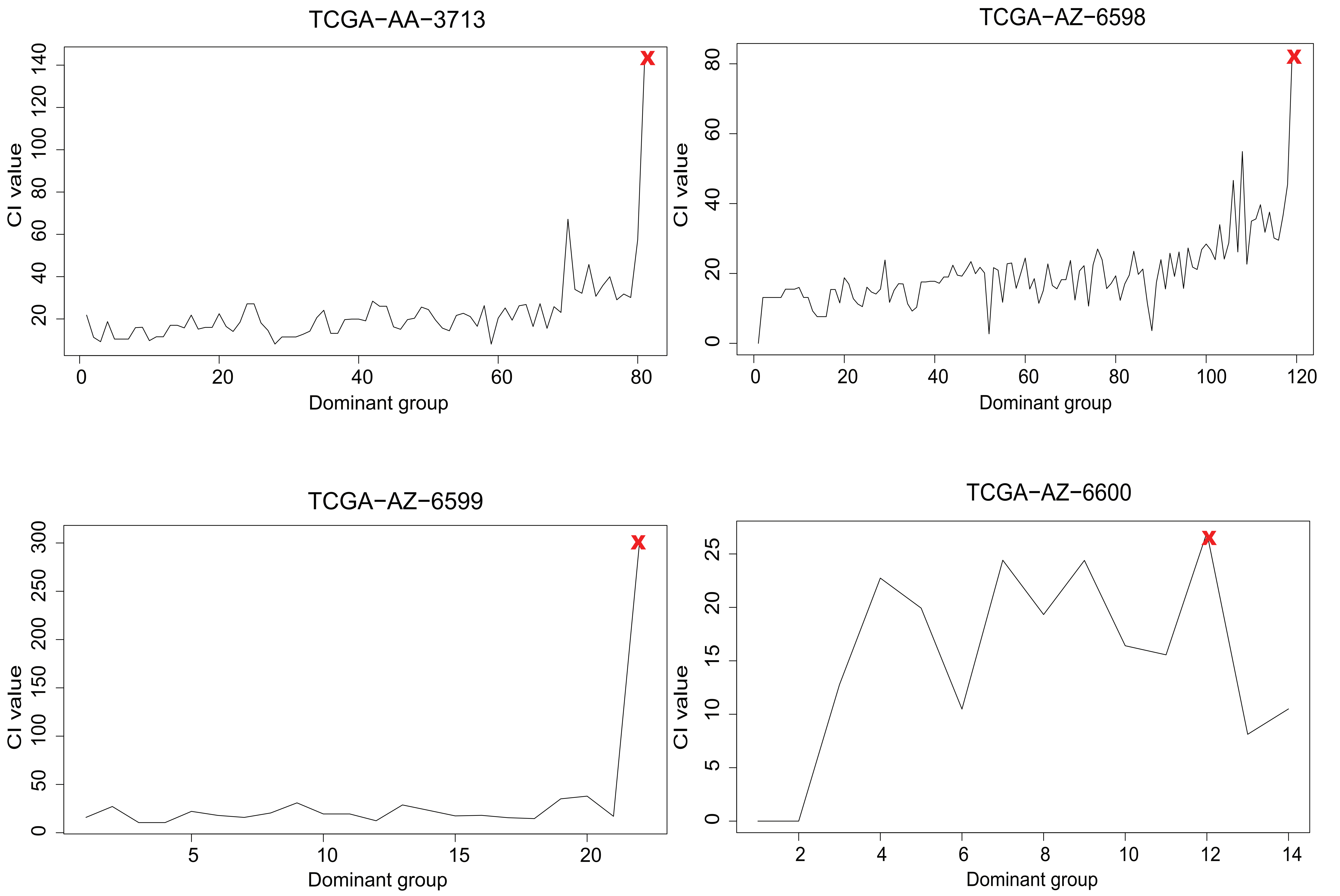

TCGA−AA−3713
TCGA−AZ−6598
TCGA−AZ−6599
TCGA−AZ−6600
CI value
Dominant group
x

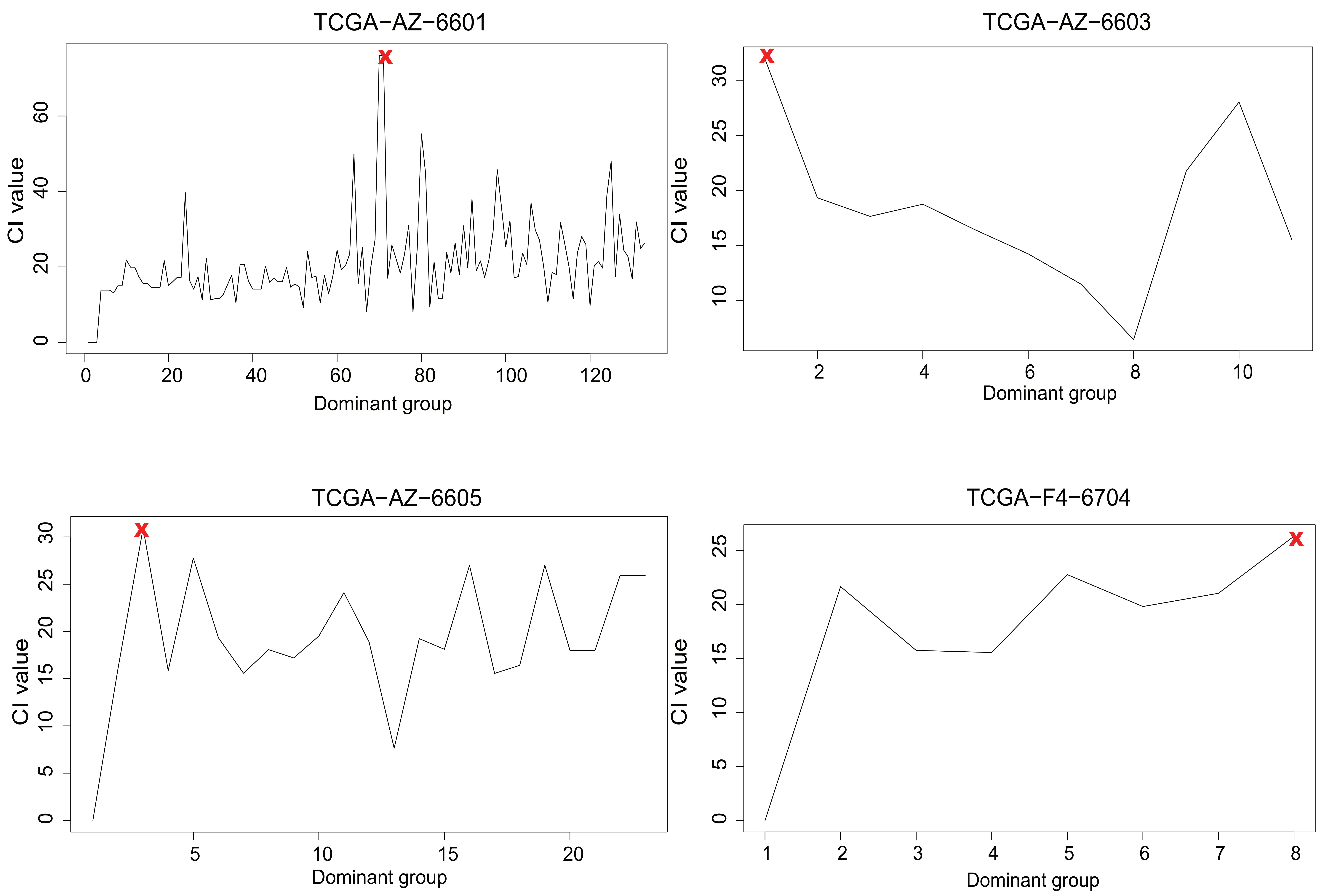

TCGA−AZ−6601
CI value
Dominant group
0
20
40
60
80
100
120
TCGA−AZ−6603
CI value
Dominant group
2
4
6
8
10
15
20
25
30
TCGA−AZ−6605
CI value
Dominant group
0
5
10
15
20
25
30
TCGA−F4−6704
CI value
Dominant group
1
2
3
4
5
6
7
8
